# BioTriangle: a web-accessible platform for generating various molecular representations for chemicals, proteins, DNAs/RNAs and their interactions

**DOI:** 10.1186/s13321-016-0146-2

**Published:** 2016-06-21

**Authors:** Jie Dong, Zhi-Jiang Yao, Ming Wen, Min-Feng Zhu, Ning-Ning Wang, Hong-Yu Miao, Ai-Ping Lu, Wen-Bin Zeng, Dong-Sheng Cao

**Affiliations:** School of Pharmaceutical Sciences, Central South University, Changsha, People’s Republic of China; College of Chemistry and Chemical Engineering, Central South University, Changsha, People’s Republic of China; School of Mathematics and Statistics, Central South University, Changsha, People’s Republic of China; School of Public Health, University of Texas Health Science Center, Houston, TX USA; Institute for Advancing Translational Medicine in Bone and Joint Diseases, School of Chinese Medicine, Hong Kong Baptist University, Hong Kong, SAR People’s Republic of China

**Keywords:** Molecular descriptors, Molecular representation, Interaction features, Online descriptor calculation, QSAR/QSPR, Cheminformatics

## Abstract

**Background:**

More and more evidences from network biology indicate that most cellular components exert their functions through interactions with other cellular components, such as proteins, DNAs, RNAs and small molecules. The rapidly increasing amount of publicly available data in biology and chemistry enables researchers to revisit interaction problems by systematic integration and analysis of heterogeneous data. Currently, some tools have been developed to represent these components. However, they have some limitations and only focus on the analysis of either small molecules or proteins or DNAs/RNAs. To the best of our knowledge, there is still a lack of freely-available, easy-to-use and integrated platforms for generating molecular descriptors of DNAs/RNAs, proteins, small molecules and their interactions.

**Results:**

Herein, we developed a comprehensive molecular representation platform, called BioTriangle, to emphasize the integration of cheminformatics and bioinformatics into a molecular informatics platform for computational biology study. It contains a feature-rich toolkit used for the characterization of various biological molecules and complex interaction samples including chemicals, proteins, DNAs/RNAs and even their interactions. By using BioTriangle, users are able to start a full pipelining from getting molecular data, molecular representation to constructing machine learning models conveniently.

**Conclusion:**

BioTriangle provides a user-friendly interface to calculate various features of biological molecules and complex interaction samples conveniently. The computing tasks can be submitted and performed simply in a browser without any sophisticated installation and configuration process. BioTriangle is freely available at http://biotriangle.scbdd.com.

**Graphical abstract:**
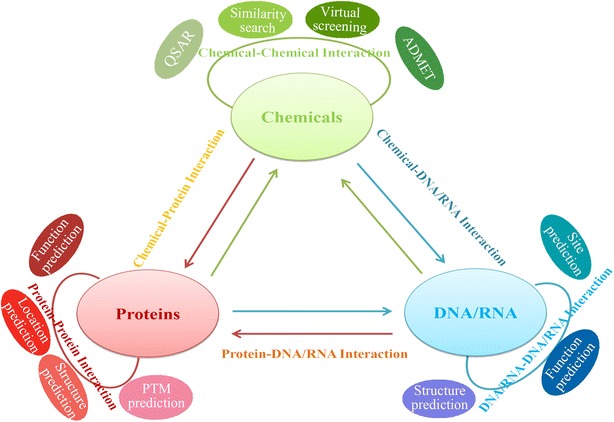
An overview of BioTriangle. A platform for generating various molecular representations for chemicals, proteins, DNAs/RNAs and their interactions

**Electronic supplementary material:**

The online version of this article (doi:10.1186/s13321-016-0146-2) contains supplementary material, which is available to authorized users.

## Background

Despite the indisputable success of the reductionism approaches in advancing our knowledge and understanding of individual molecules and their functions, it has been increasingly recognized that a single biological process often involves complex interactions among a variety of molecules, especially DNA, RNA, proteins and small molecules [1, 2]. Systematic investigation and understanding of human interactome (i.e., complex networks resulted from numerous interactions among nucleotides, proteins, metabolites etc.) is thus becoming a key research area, which could fundamentally renovate our thinking on how to develop novel and more efficient therapeutic or preventive interventions (e.g., the network medicine concept) [1, 3].

In previous studies, particular attention has been paid to a variety of molecular interaction networks and their potential roles in disease mechanism and drug development [1, 4–7], including transcriptional and post-transcriptional regulatory networks [8–10], functional RNA networks [11–13], protein–protein interaction networks [14, 15], and metabolic networks [16, 17]. Consequently, public databases for human-specific molecular interaction data have been undergoing a rapid growth within the past decade, such as BIND [18], DIP [19], STITCH [20], HPRD [21], TTD [22], DrugBank [23], ChEMBL [24], KEGG [25], BindingDB [26], SuperTarget and Matador [27], to name a few. However, the heterogeneity of data in such databases poses a significant challenge to their integration and analysis in practice. In particular, the bioinformatics and the cheminformatics communities have evolved more or less independently, e.g., with an emphasis on macro biomolecules and chemical compounds, respectively. However, to investigate complex molecular interactions, both biological and chemical knowledge on structures and functions of all the involved molecules are required, especially in the scenarios of identifying new drug targets and their potential ligands or discovering potential biomarkers for complex diseases [28–30]. Therefore, it is necessary and useful to build informatics platforms for unified data or knowledge representation that can integrate the existing efforts from both communities.

Molecular descriptors are one of the most powerful approaches to characterize the biological, physical, and chemical properties of molecules and have long been used in various studies for understanding molecular interactions or drug development [31–34]. In the bioinformatics and cheminformatics fields, sequence- and structure-based constitutional, physicochemical, and topological features have been widely used in the development of computing algorithms for predicting protein structural and functional classes [29], protein–protein interactions [35], subcellular locations and peptides of specific properties [36], drug-target pairs and associations [37, 38], meiotic recombination hot spots [39], and nucleosome positioning in genomes [40], etc. Besides its capability of describing and distinguishing nucleotides, proteins and small molecules of different structural, functional and interaction profiles, we further stress that molecular descriptor provides a convenient and consistent way of molecular or interaction representation, and is thus a suitable choice to meet the needs mentioned in the previous paragraph.

Several bioinformatics packages for computing structural and physicochemical features of proteins or DNAs/RNAs have been previously developed, including PROFEAT [41], BioJava [42], PseAAC, propy [43], repDNA [44], repRNA [45], protr [46] etc. In the cheminformatics field, several open sources or commercial software for drug discovery (e.g., QSAR/SAR [47], virtual screening [48], database search [49], drug ADME/T prediction [50, 51]) have been implemented for computing molecular descriptors of small molecules, including Dragon, CODESSA, Chemistry Development Kit (CDK) [52], chemopy [53], Molconn-Z, OpenBabel [54], Cinfony [55], Rcpi [56], Indigo, JOELib, Avogadro and RDKit. However, all the tools mentioned above only support a limited number of molecular types or descriptors, and they may not be freely available or easily accessible. To the best of our knowledge, there is still a lack of freely-available and integrated platforms for generating molecular descriptors of DNA/RNA, proteins, small molecules and their interactions [57].

In this study, we develop a comprehensive molecular representation tool, called BioTriangle, for characterizing various complex biological molecules and pairwise interactions. More specifically, BioTriangle can calculate a large number of molecular descriptors of chemicals from their topology, structural and physicochemical features of proteins and peptides from their amino acid sequences, and composition and physicochemical features of DNAs/RNAs from their primary sequences. Furthermore, BioTriangle can calculate the interaction descriptors between two individual molecules. To ease the use of the BioTriangle utilities and functionalities, we also provide users a friendly and uniform web interface. For illustration purpose, we use five datasets from different applications as representative examples to show how BioTriangle can be used in an analytical pipeline. We thus recommend BioTriangle when molecular or interaction representation is need for exploring questions concerning structures, functions and interactions of various molecular data in the context of biomedical studies.

## Implementation and user interface

BioTriangle is designed as a web application implemented in an open source Python framework (Django) for the Graphical User Interface (GUI) and MySQL for data retrieval. The Nginx + uWSGI architecture is used to enable an efficient data exchange between dynamic data from the server-side and static contents form client-side. By employing this architecture, the balance between system resource occupation and computational efficiency is maintained; the good independence and safety of a long time data operation and file access from different requests are also guaranteed. The JavaScript and jQuery were employed to help accomplish some complex interaction processes, result visualization and download at front-side. Pybel, a Python wrapper of the OpenBabel [54] toolkit, was used in backend for chemical structure parsing and converting. CSB.bio [58], a Python package which provides plenty of APIs for bioinformatics was used in backend for protein and DNAs/RNAs sequence parsing. The main calculation procedures and transaction processing procedures are written in Python language.

To provide an online computing service based on web, the user interface should be convenient and easy-to-use for users. In the following paragraph, we briefly describe the user interface of the BioTriangle. The user interface of BioTriangle consists of six main modules: “Home”, “Webserver”, “Documentation”, “Tools”, “Tutorials” and “FAQ”. In the “Home” module, a summary of the platform and the quick-start entrance of each tool are provided to users. This gives users a clear understanding of the platform and a better selection of the tools. The “Webserver” module is the main entrance for users to choose different tools to perform their calculation. The “Documentation” module provides detailed definitions and references of the descriptors form each tool, so that users can look for the detailed information of certain descriptors conveniently. Besides, it also provides the usages of all the tools that users can use them quickly and easily. In the “Tools” module, several Python scripts for specific functionalities are available for better use of the platform. In the “Tutorials” module, five typical applications by using BioTriangle are listed there and all the related data are available for download. The “FAQ” module provides some frequently asked questions and the solutions are also listed there.

## Methods and results

### BioTriangle overview

**Fig. 1:**
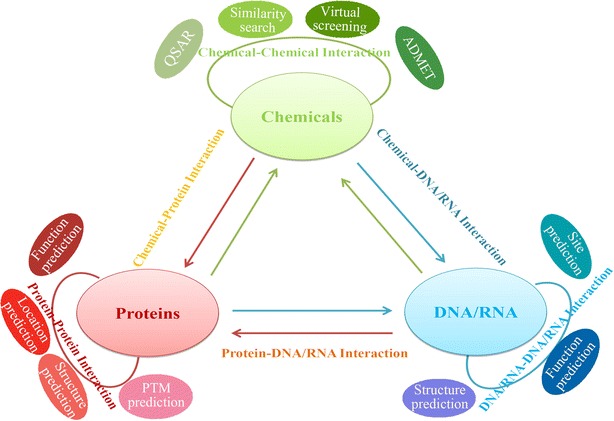
The overview of *BioTriangle* web server. *BioTriangle* could calculate various molecular descriptors from chemicals, proteins, DNAs/RNAs and their interactions

As its name denoted, BioTrianlge constructs the interaction between any two molecular objects in terms of the features from three main molecular types (see Fig. 1). Nine individual tools in BioTriangle correspond to the calculation of nine types of molecular features. Of these, BioChem, BioProt, and BioDNA are corresponding to the calculation of chemicals, proteins and DNAs/RNAs, respectively. BioCCI, BioPPI, and BioDDI are corresponding to the calculation of chemical–chemical interaction, protein–protein interaction, and DNA/RNA-DNA/RNA interaction, respectively. Likewise, BioCPI, BioDPI, and BioCDI are corresponding to the calculation of chemical–protein interaction, DNA/RNA–protein interaction, and chemical–DNA/RNA interaction, respectively. The detailed instructions for molecular features and how to use these tools are provided in the documentation section of the platform. The users can select the corresponding tools to calculate the features as needed.

In addition to main functionalities mentioned above, BioTriangle can also provide a number of supplementary functionalities to facilitate the computation of molecular features. To obtain different biological molecules easily, BioTriangle provides four Python scripts in the tool section, with which the user could easily get molecular structures or sequences from the related websites by providing IDs or a file containing IDs. This greatly facilitates the acquisition of different molecules for users. Moreover, BioTriangle also provides a BioModel script to construct the prediction models based on the data matrix generated by BioTriangle. The users could select different machine learning methods to construct their models as needed.

### Molecular descriptors from chemical structures

**Fig. 2:**
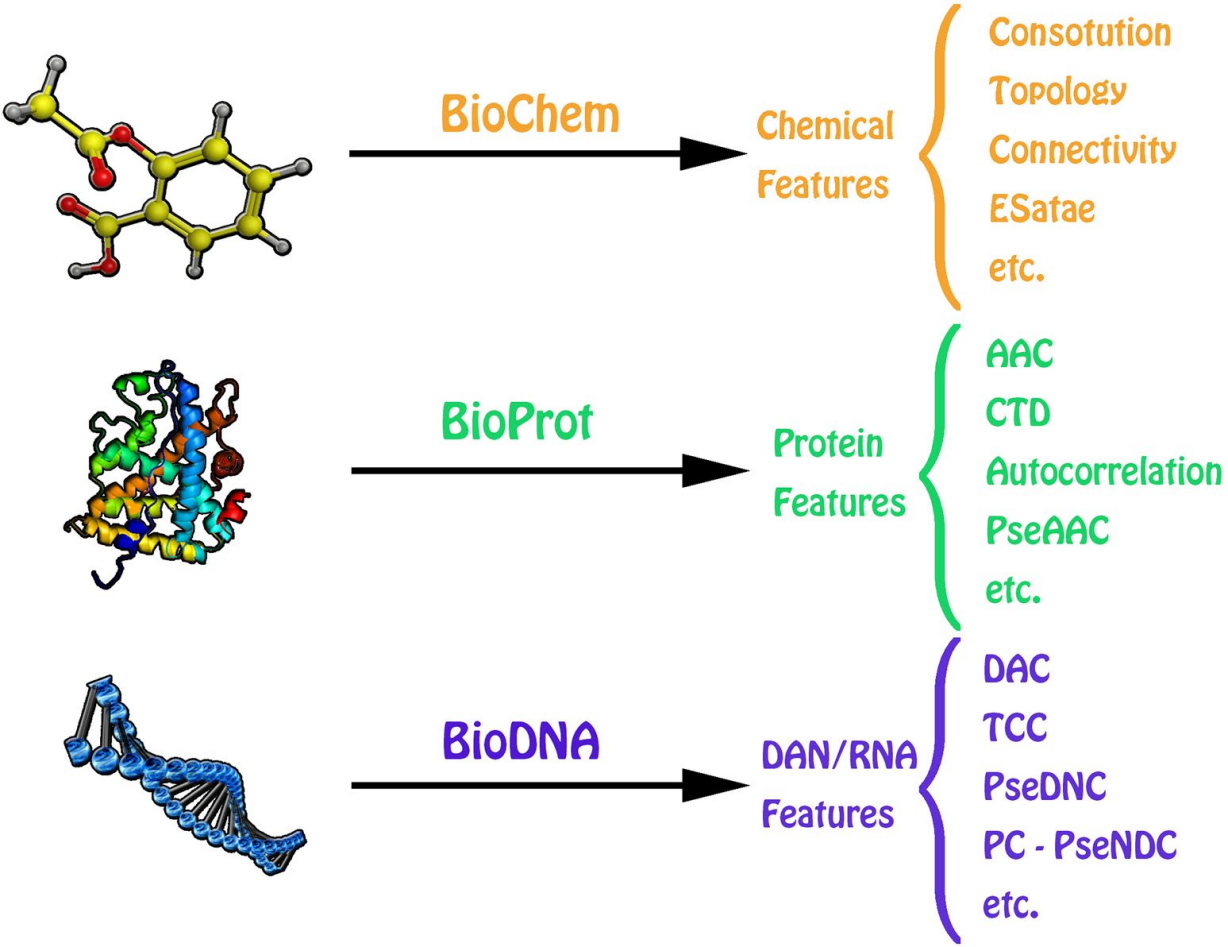
The schematic diagram of single molecular descriptor calculation. Molecular features from chemicals, proteins, and DNAs/RNAs could be easily calculated through BioChem tool, BioProt tool and BioDNA tool, respectively

Nine groups of molecular descriptors are calculated to represent small molecules in BioChem. A detailed list of small molecular descriptors covered by BioChem is summarized in Table 1. These descriptors capture and magnify distinct aspects of chemical structures. The usefulness of molecular descriptors in the representation of molecular information is reflected in their widespread adoption and use across a broad range of applications and methodologies, as reported in a large number of published articles. The users could select one or more groups to represent the chemicals under investigation (see Fig. 2).

**Table 1:** List of BioChem computed features for chemical molecules

Feature group	Features	Number of descriptors
Constitution	Molecular constitutional descriptors	30
Topology	Topological descriptors	35
Connectivity	Molecular connectivity indices	44
E-state	E-state descriptors	237
Kappa	Kappa shape descriptors	7
Autocorrelation	Moreau-Broto autocorrelation	32
	Moran autocorrelation	32
	Geary autocorrelation	32
Charge	Charge descriptors	25
Property	Molecular property	6
MOE-type	MOE-type descriptors	60
Fingerprints	Topological fingerprints	2048
	MACCS keys	166
	FP4 keys	307
	E-state fingerprints	79
	Atom pairs fingerprints	–
	Topological torsions	–
	Morgan fingerprints	–

Constitutional descriptors consist of 30 descriptor values, which are mainly used for characterizing the composition of chemical element type and chemical bond type, path length, hydrogen bond acceptor, and donator in the constitution module. Topology descriptors are those invariants calculated from molecular topological structure, which have been successfully used for predicting molecular physicochemical properties, such as boiling point and retention index etc. In the topology group, 35 commonly used topological descriptors like Weiner index, Balaban index, Harary index, and Schultz index are computed. Molecular connectivity indices consist of 44 descriptor values that reflect simple molecular connectivity and valence connectivity for different path orders, cycle, or cluster size. They are among the most popular indices and are calculated from the vertex degree of the atoms in the H-depleted molecular graph. The connectivity group is responsible for the calculation of all connectivity descriptors. Kappa shape indices are computed through the kappa group, each of which represents a particular shape attribute of the molecule, such as molecular flexibility, molecular steric effect, molecular symmetry etc. Seventy-nine atom-type E-state indices were proposed in the estate group as molecular descriptors encoding topological and electronic information related to particular atom types in the molecule. E-state indices are especially useful in the prediction of drug ADME/T. In addition, the maximum and minimum of E-state values of 79 atom types are also calculated as molecular descriptors in BioChem. Six commonly used molecular properties are directly used in the molecular property group for representing the molecule, including molar refractivity, LogP based on Crippen method and its square, topological polarity surface area, unsaturation index, and hydrophilic index. Twenty-five charge descriptors are computed based on Gasteiger–Marseli partial charges in the charge group, which describe electronic aspects both of the whole molecule and of particular regions, such as atoms, bonds, and molecular fragments. Electrical charges in the molecule are the driving force of electrostatic interactions and it is well known that local electron densities or charges play a fundamental role in many chemical reactions, physicochemical properties, and receptor-ligand binding. Three types of autocorrelation descriptors (i.e., Moreau-Broto, Moran, Geary) are computed in the three individual group, respectively. Four carbon-scaled atomic properties are used to calculate these descriptors, including atomic mass, atomic Van der Waals volume, atomic Sanderson electronegativity, and atomic polarizability. Sixty MOE-type descriptors can be computed from connection table information based on atomic contributions to Van der Waals surface area, LogP, molar refractivity, partial charge, and E-state value. These descriptors have been frequently applied to the construction of QSAR models for boiling point, vapor pressure, thrombin/factor Xa activity, blood–brain barrier permeability, and compound classification. All functionalities used for computing MOE-type descriptors are included in the MOE-type descriptor group. The detailed definition and description of each molecular descriptor could be found in the documentation section of the website (see Additional file 1).

Another striking feature in BioChem is the computation of a number of molecular fingerprints. Molecular fingerprints are string representations of chemical structures, which consist of bins, each bin being a substructure descriptor associated with a specific molecular feature. Seven types of molecular fingerprints are provided in BioChem, including topological fingerprints, E-state fingerprints, MACCS keys, FP4 keys, atom pairs fingerprints, topological torsion fingerprints, and Morgan/circular fingerprints. The usefulness of these molecular fingerprints covered by BioChem have been sufficiently demonstrated by a number of published studies of the development of machine learning classification systems in QSAR/SAR, drug ADME/T prediction, similarity searching, clustering, ranking and classification.

### Protein or peptide descriptors from amino acid sequences

A list of features for proteins and peptides covered by BioProt is summarized in Table 2. These features can be divided into six groups, each of which has been independently used for predicting protein- and peptide-related problems by using machine learning methods (see Fig. 2). More detailed description and references can be found in the documentation section from BioTriangle (see Additional file 2).

**Table 2:** List of BioProt computed features for protein sequences

Feature group	Features	Number of descriptors
Amino acid composition	Amino acid composition	20
	Dipeptide composition	400
	Tripeptide composition	8000
Autocorrelation	Normalized Moreau–Broto autocorrelation	240^a^
	Moran autocorrelation	240^a^
	Geary autocorrelation	240^a^
CTD	Composition	21
	Transition	21
	Distribution	105
Conjoint triad	Conjoint triad features	343
Quasi-sequence order	Sequence order coupling number	60
	Quasi-sequence order descriptors	100
Pseudo amino acid composition	Pseudo amino acid composition	50^b^
	Amphiphilic pseudo amino acid composition	50^c^

^a^The number depends on the choice of the number of properties of amino acid and the choice of the maximum values of the *lag*. The default is eight types of properties and *lag* = 30

^b^The number depends on the choice of the number of the set of amino acid properties and the choice of the *λ* value. The default is three types of properties proposed by Chou et al. and *λ* = 30

^c^The number depends on the choice of the *λ* value. The default is that *λ* = 15

The first group includes three features: amino acid composition, dipeptide composition and tripeptide composition, with 3 descriptors and 8420 descriptor values. These descriptors represent the fraction of each amino acid type, dipeptide type and tripeptide type in a protein sequence. These simplistic descriptors can be used to predict protein fold and structural classes, functional classes, and subcellular locations.

The second group consists of three different autocorrelation features: normalized Moreau–Broto autocorrelation, Moran autocorrelation, and Geary autocorrelation. The autocorrelation features describe the level of correlation between two protein or peptide sequences in terms of their specific structural or physicochemical property. In the default settings, there are eight amino acid properties used for deriving these autocorrelation descriptors. Thus, three autocorrelation features are computed, each having 8 descriptors and 8 × 30 = 240 descriptor values. Autocorrelation descriptors can be used for predicting transmembrane protein types, protein helix contents, and protein secondary structural contents.

The third group contains three feature sets: composition (C), transition (T), and distribution (D), with a total of 3(C) + 3(T) + 5 × 3(D) = 21 descriptors, and 147 descriptor values. They represent the amino acid distribution pattern of a specific structural or physicochemical property along a protein or peptide sequence. Seven types of physicochemical properties have been used for calculating these features, including hydrophobicity, polarity, charge, polarizibility, normalized Van der Waals volume, secondary structures, and solvent accessibility. C is the number of amino acids of a particular property (e.g., hydrophobicity) divided by the total number of amino acids in a protein sequence. T characterizes the percent frequency with which amino acids of a particular property is followed by amino acids of a different property. D measures the chain length within which the first, 25, 50, 75, and 100 % of the amino acids of a particular property are located, respectively. These CTD features have been widely used for predicting protein folds [59], protein–protein interactions [60], and protein functional families [61] at accuracy levels of 74–100, 77–81, 67–99 %, respectively.

The fourth group, conjoint triad descriptors, proposed by Shen et al. [35], was originally designed to represent protein–protein interactions. These conjoint triad features abstract the features of protein pairs based on the classification of amino acids. Twenty amino acids were clustered into several classes according to their dipoles and volumes of side chains. Herein, the dipoles and volumes of side chains of amino acids, reflecting electrostatic and hydrophobic interactions, were calculated, respectively, by using the density-functional theory method B3LYP/6-31G* and molecular modeling approach. The reason for dividing amino acids into seven groups is that amino acids within the same class likely involve synonymous mutations because of their similar characteristics. The conjoint triad features consider the properties of one amino acid and its neighboring ones and regard any three continuous amino acids as a unit. Thus, the triads can be differentiated according to the classes of amino acids, i.e., triads composed by three amino acids belonging to the same classes could be treated identically. For amino acids that have been catalogued into seven classes, we can finally construct a 7 × 7 × 7 = 343-dimensional vector, each dimension of which records the frequency of each triad appearing in the protein sequence. For detailed information on how to calculate these features, please refer to the documentation section of the website. Applying the conjoint triad features to the prediction of protein–protein interactions, the support vector machine based on S-kernel function obtained an average prediction accuracy of 83.90 % on test sets [35].

The fifth group includes two sequence-order feature sets, one is sequence-order-coupling number with 2 descriptors and 60 descriptor values, and the other is quasi-sequence-order with 2 descriptors and 100 descriptor values. These features are derived from both Schneider–Wrede physicochemical distance matrix and Grantham chemical distance matrix. The sequence-order features can be used for representing amino acid distribution patterns of a specific physicochemical property along a protein or peptide sequence, which have been used for predicting protein subcellular locations.

The sixth group contains two types of pseudo-amino acid compositions (PseAAC): type I PseAAC with 50 descriptor values and type II PseAAC (i.e., amphiphilic PseAAC) with 50 descriptor values. In simple amino acid composition, all the sequence-order effects are missing. To avoid losing the sequence-order information completely, the concept of PseAAC, developed by K.C. Chou, was mainly used to reflect the composition of amino acids and the sequence-order information (at least partially) through a set of correlation factors. PseAAC has been frequently used in improving the prediction quality for subcellular location of proteins and their other attributes.

### DNA/RNA descriptors from nucleotide sequences

Generally, three groups of features from nucleotide sequences are calculated to represent DNA/RNA in BioDNA (see Fig. 2). A detailed list of descriptors for DNA/RNA covered by BioDNA is summarized in Table 3. The usefulness of these features covered by BioDNA for representing DNA/RNA sequence information have been sufficiently demonstrated by a number of published studies of the development of machine learning classification systems in computational genomics and genome sequence analysis. More detailed description and references can be found in the documentation section of the website (see Additional file 3).

**Table 3:** List of BioDNA computed features for DNA/RNA sequences

Feature group	Features	Number of descriptors^a^
Nucleic acid composition	Basic kmer	16
	Reverse compliment kmer	10
Autocorrelation	Dinucleotide-based auto covariance	74
	Dinucleotide-based cross covariance	2664
	Dinucleotide-based auto-cross covariance	2738
	Trinucleotide-based auto covariance	24
	Trinucleotide-based cross covariance	264
	Trinucleotide-based auto-cross covariance	288
Pseudo nucleic acid composition	Pseudo dinucleotide composition	18
	Pseudo k-tuple nucleotide composition	18
	Parallel correlation pseudo dinucleotide composition	18
	Parallel correlation pseudo trinucleotide composition	66
	Series correlation pseudo dinucleotide composition	90
	Series correlation pseudo trinucleotide composition	88

^a^The number depends on the choice of the values of the parameters in the formula. Here, the number of each type of descriptors is based on the default parameter value. For detailed information, please refer to the documentation section in the BioTriangle website

The first group includes two features: basic kmer and reverse compliment kmer. Basic kmer is the simplest approach to represent the DNAs, in which the DNA sequences are represented as the occurrence frequencies of k neighboring nucleicacids. The reverse compliment kmer is a variant of the basic kmer, in which the kmers are not expected to be strand-specific, so reverse complements are collapsed into a single feature. For more information of this approach, please refer to Gupta et al. [62] and Noble et al. [63]. These simplistic descriptors have been successfully applied to human gene regulatory sequence prediction, enhancer identification, etc.

The second group consists of six different autocorrelation features. Autocorrelation, as one of the multivariate modeling tools, can transform the DNA sequences of different lengths into fixed-length vectors by measuring the correlation between any two properties. Autocorrelation results in two kinds of variables: autocorrelation (AC) between the same property, and cross-covariance (CC) between two different properties. Herein, BioDNA allows users to calculate various kinds of autocorrelation feature vectors for given DNA sequences or FASTA files by selecting different methods and parameters. BioDNA aims at computing six types of autocorrelation, including dinucleotide-based auto covariance (DAC), dinucleotide-based cross covariance (DCC), dinucleotide-based auto-cross covariance (DACC), trinucleotide-based auto covariance (TAC), trinucleotide-based cross covariance (TCC), and trinucleotide-based auto-cross covariance (TACC). Autocorrelation features exhibit good prediction performance in the mammalian enhancers, human transcription start site, splice site, and so on.

The third group is the pseudo nucleic acid composition (PseNAC) features. PseNAC represents the DNA sequences considering both DNA local sequence-order information and long range or global sequence-order effects. Herein, BioDNA aims at computing six types of pseudo nucleic acid composition: pseudo dinucleotide composition (PseDNC), pseudo k-tuplenucleotide composition (PseKNC), parallel correlation pseudo dinucleotidecomposition (PC-PseDNC), parallel correlation pseudo trinucleotide composition (PC-PseTNC), series correlation pseudo dinucleotide composition (SC-PseDNC), and series correlation pseudo trinucleotide composition (SC-PseTNC). The users could calculate various kinds of PseNAC feature vectors for given DNA sequences or FASTA files by selecting different methods and parameters. The usefulness of PseDNC related features has been well demonstrated in improving the prediction quality for nucleosome positioning in genomes, recombination spots, human nucleosome occupancy, and so on.

### Descriptors from the interaction between two molecules with the same type

**Fig. 3:**
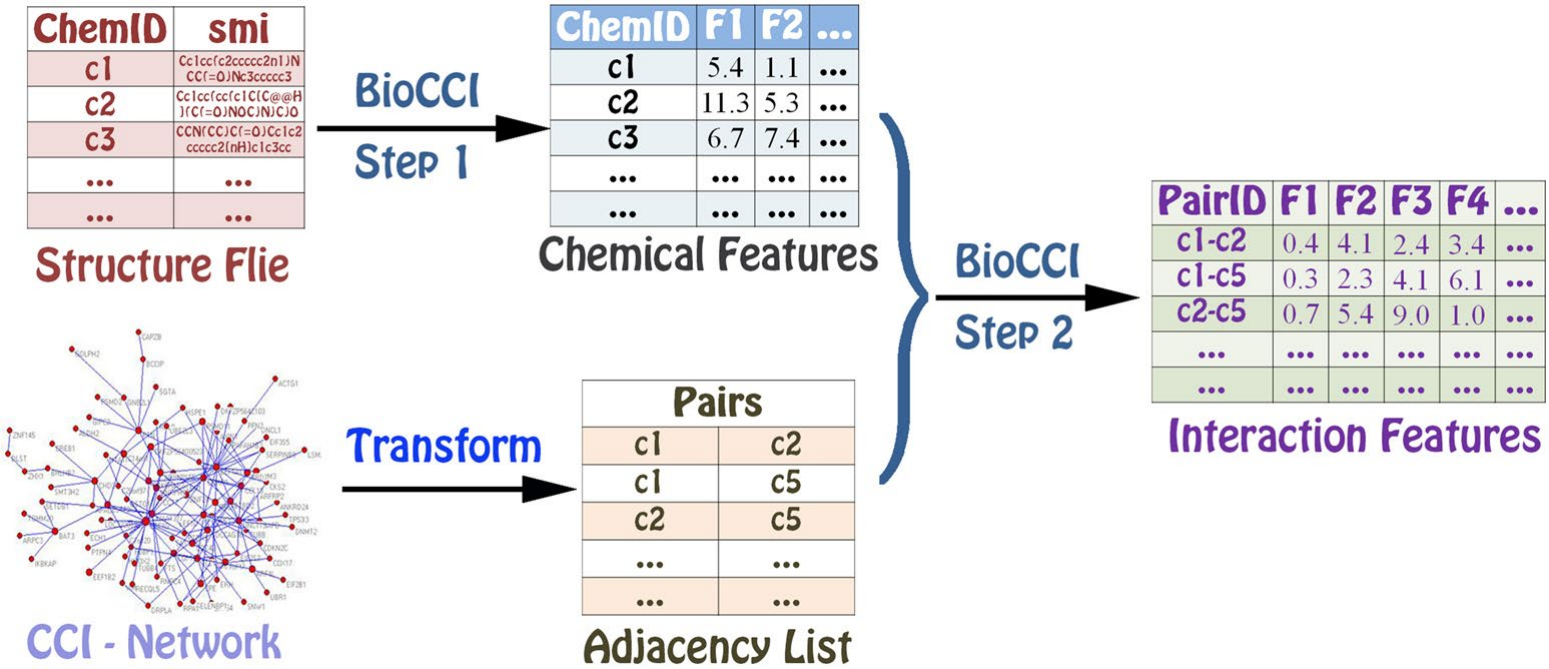
The schematic diagram of descriptor calculation from the interaction between two molecules with the same type. The calculation process for BioCCI, BioPPI and BioDDI is similar to each other. Firstly, the molecular structures or sequences of the associated chemicals, proteins, and DNAs/RNAs in the chemical-chemical, protein–protein, and DNA/RNA–DNA/RNA interaction networks are provided to calculate the corresponding molecular features. Secondly, the adjacency list file and the molecular features in the above step are provided to calculate the final interaction features

The descriptor calculation of chemical–chemical interaction, protein–protein interaction, and DNA/RNA-DNA/RNA interaction is similar to each other in BioCCI, BioPPI and BioDDI (see Additional file 4). We will show how to construct an interaction feature by the protein–protein interaction example (see Fig. 3). Let **F**_**a**_ = {**F**_**a**_(i), i = 1, 2, …, p} and **F**_**b**_ = {**F**_**b**_(i), i = 1, 2, …, p} are the two descriptor vectors for interaction protein A and protein B, respectively. There are three methods to construct the interaction descriptor vector **F** for A and B:

1. Two vectors **F**_**ab**_ and **F**_**ba**_ with dimension of 2p are constructed: **F**_**ab**_ = (**F**_**a**_, **F**_**b**_) for interaction between protein A and protein B and **F**_**ba**_ = (**F**_**b**_, **F**_**a**_) for interaction between protein B and protein A.
2. One vector **F** with dimension of 2p is constructed: **F** = {**F**_**a**_(i) + **F**_**b**_(i), **F**_**a**_(i) × **F**_**b**_(i), i = 1, 2, …,p}.
3. One vector **F** with dimension of p^2^ is constructed by the tensor product: **F** = {**F**(k) = **F**_**a**_(i) × **F**_**b**_(j), i = 1, 2, …, p, j = 1, 2,…, p, k = (i − 1) × p + j}.

### Descriptors from the interaction between two molecules with different types

**Fig. 4:**
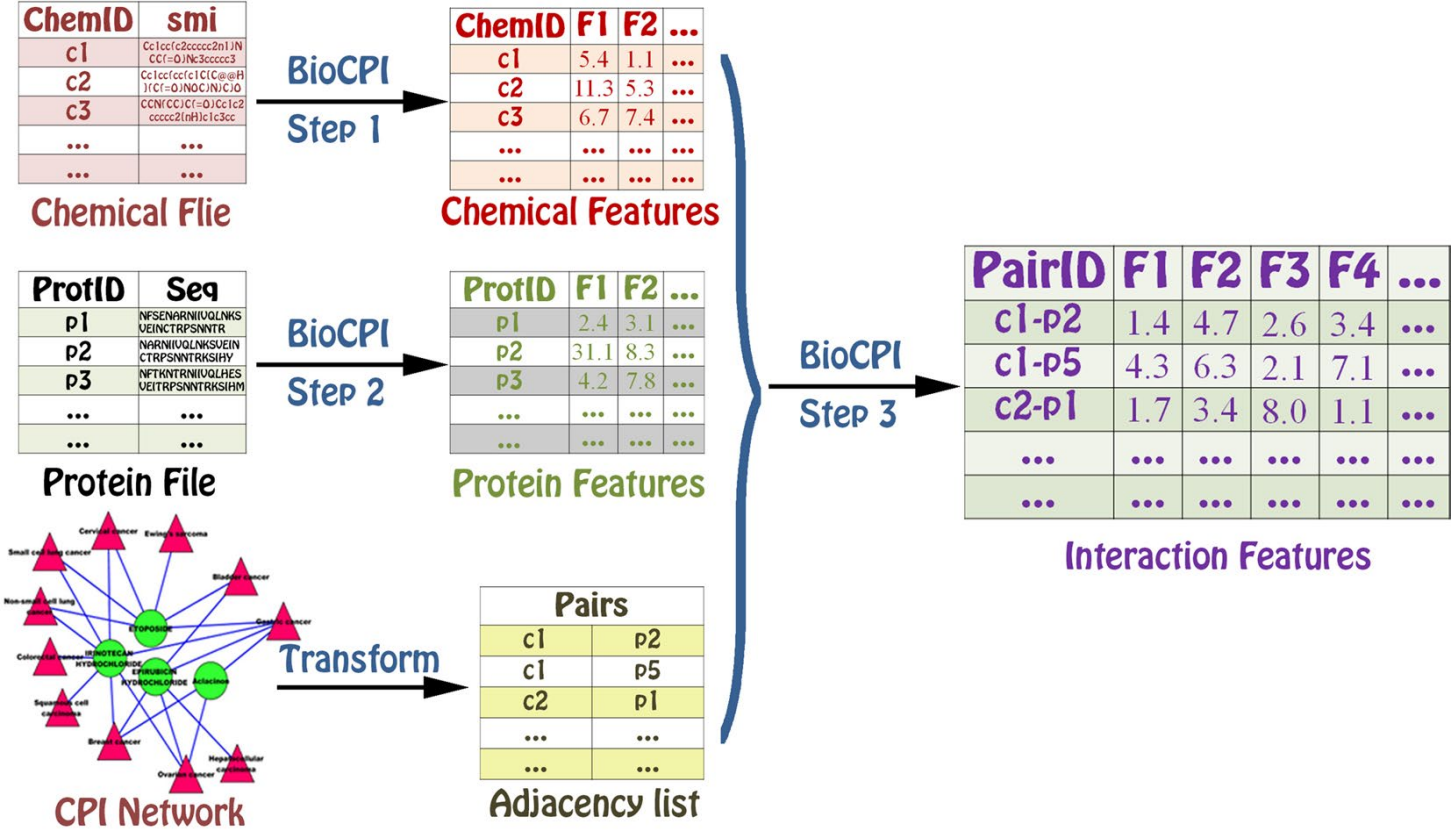
The schematic diagram of descriptor calculation from the interaction between two molecules with the different types. The calculation process for BioCDI, BioCPI and BioDPI is similar to each other. Firstly, molecular structures or sequences of the associated chemicals, proteins, and DNAs/RNAs in the chemical–chemical, protein–protein, and DNA/RNA–DNA/RNA interaction networks are provided to calculate the corresponding molecular features. Secondly, the molecular structures or sequences of another molecular object from interaction data are provided to calculate the corresponding molecular features. Thirdly, the adjacency list file and the molecular features in the above two steps are provided to calculate the final interaction features

The descriptor calculation of chemical-protein interaction, protein-DNA/RNA interaction, and chemical-DNA/RNA interaction is similar to each other in BioCPI, BioDPI and BioCDI (see Additional file 5). Likewise, we will show how to construct an interaction feature by the chemical-protein interaction example (see Fig. 4). There are two methods for construction of descriptor vector **F** for chemical-protein interaction from the protein descriptor vector **F**_**t**_ (**F**_**t**_(i), i = 1, 2, …, p_t_) and chemical descriptor vector **F**_**d**_ (**F**_**d**_(i), i = 1, 2, …, p_d_):

1. One vector **V** with dimension of p_t_ + p_d_ is constructed: **F** = (**F**_**t**_, **F**_**d**_**)** for interaction between protein t and chemical d.
2. One vector **V** with dimension of p_t_ × p_d_ is constructed by the tensor product: **F** = {**F**(k) = **F**_**t**_(i) × **F**_**d**_(j), i = 1, 2, …, p_t_, j = 1, 2, …, p_d_, k = (i − 1) × p_t_ + j}.

### Input/output

**Fig. 5:**
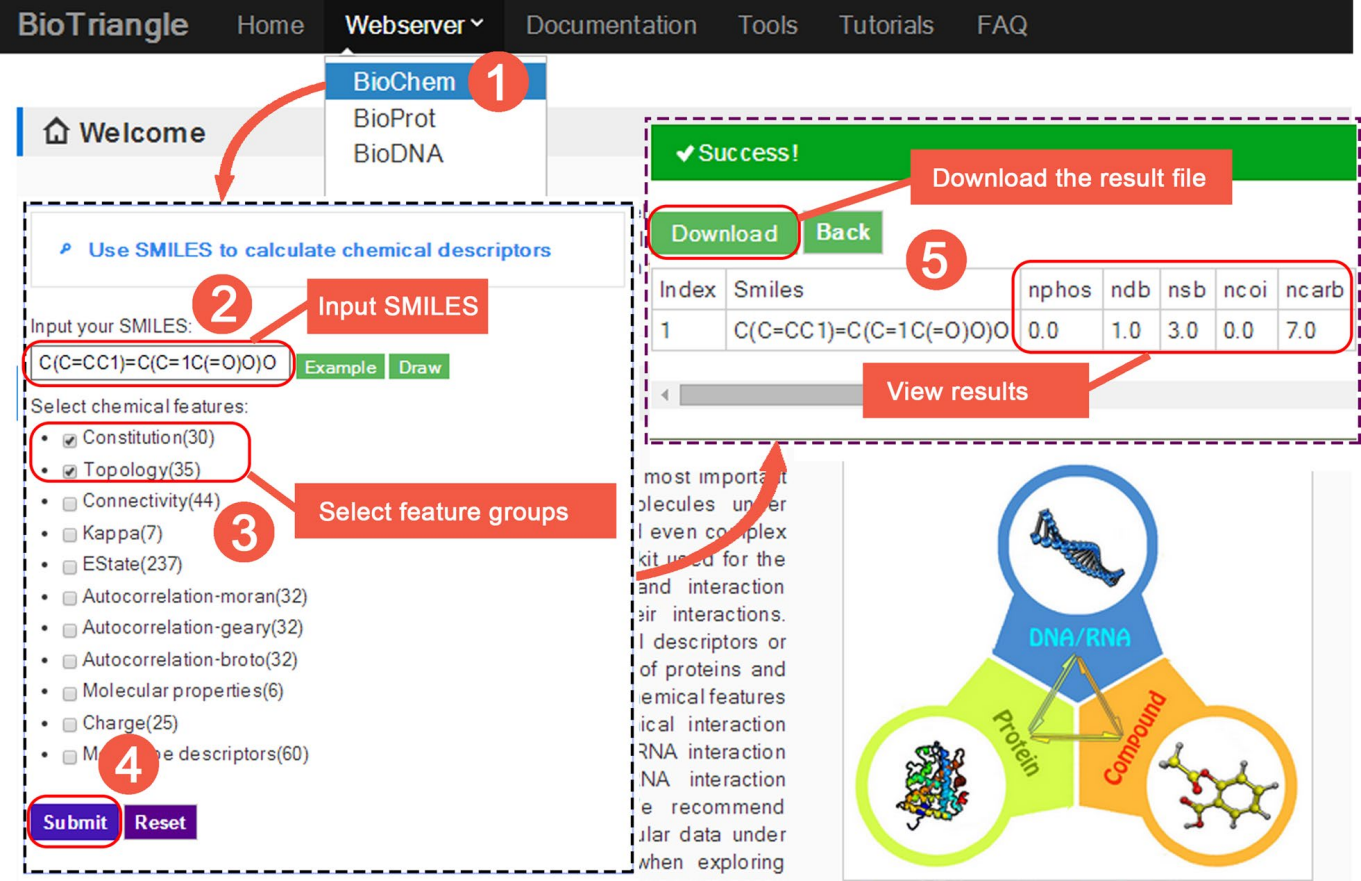
A screenshot of the web interface by using BioChem tool. To use BioChem, users should firstly go to the index page (marked number *1* in the picture). Then, input molecules and choose feature groups (marked number *2*, *3*, and *4*). After submitting, calculation results will be displayed in the result page (marked number *5*)

BioTriangle consists of nine tools. Each of them accepts a string or a file as uniform input and then collects the calculation results to users at the result page. There, an HTML table contains results are displayed to users and a *.*CSV* file are available for download. Besides, a step-by-step strategy is applied in BioTriangle during the computing process, which makes it convenient to get an example in each step and save the calculation results in time. The Fig. 5 shows a screenshot of the web interface by using BioChem tool. BioChem accepts a *SMILES* string, *.SDF file and *.SMI file as the input; the BioProt accepts a single protein *FASTA* sequence string or protein *.FASTA file as the input; the BioDNA accepts a DNA/RNA *FASTA* sequence string or DNA/RNA *.FASTA file as the input. As for the other six tools, a tab-delimited text file (*.TXT) is acceptable as the input. This kind of format makes it easy to edit on multiple operating system platforms (Windows, Linux and Mac OS platform) and by any text editor. The detailed information about how to format the *.TXT file for each calculation step is described in the FAQ section of the website.

## Discussion

Considering the amazing rate at which data are accumulated in chemistry and biology fields, new tools that process and interpret large and complex interaction data are increasingly important. However, to our knowledge, no open source or freely available tool exists to perform these functions above. BioTriangle is a powerful web server for the extraction of features of complex interaction data. After representation, different statistical learning tools can be applied for further analysis and visualization of the data. Several case studies from wide applications show how BioTriangle was used to describe various molecular features and establish a model in a routing way (see the Tutorials section). The application domain of BioTriangle is not limited to the interaction data. It can, as Fig. 1 shows, be applied to a broad range of scientific fields such as QSAR/SAR, similarity search, absorption, distribution, metabolism, elimination and toxicity (ADMET) prediction, virtual screening, protein function/substructure/family classification, subcellular locations, post-translational modification (PTM), DNA structure/function/site prediction, and various interaction data analysis. We expect that BioTrianlge will better assist chemists, pharmacologists and biologists in characterizing, analyzing, and comparing complex molecular objects.

The current version of BioTrianlge has a number of strengths that make it useful for a wide variety of applications in computational biology. The usefulness of the features covered by BioTrianlge has been extensively tested by a number of published studies of the development of statistical learning algorithms for analyzing various biological, chemical and biomedical problems. Several web-based servers have been established to perform these tasks such as SVM-Prot [61], Cell-Ploc [36], iGPCR-Drug [64], iRSpot-PseDNC [39], IDrug-Target [65] and iNuc-PseKNC [66]. The similarity principle is prominent in medicinal chemistry, although it is well known as the similarity paradox, i.e., those very minor changes in chemical structure can result in total loss of activity. Based on different similarities, various molecular fingerprint systems were used for identifying novel drug targets. Campillos et al. proposed a novel method to identify new targets based on the similarity of side effects by Daylight-type topological fingerprints [67]. Twenty of unexpected DTIs are tested and thirteen of which are successfully validated by in vitro binding assays. A method to predict protein targets based on chemical similarity of their ligands was proposed by Keiser et al. using Daylight-type topological fingerprints and extended-connectivity fingerprints [68]. They confirmed 23 new DTIs and found that 5 ones were potent with Ki values <100 nM. A number of studies have been performed on the modeling of the interaction of GPCR with a diverse set of ligands using a proteochemometrics approach [69–71], which aims at finding an empirical relation that describes the interaction activities of the biopolymer-molecule pairs as accurately as possible, based on a unified description of the physicochemical properties of the primary amino acid sequences of proteins, and the description of the physicochemical properties of the ligands that may interact with the proteins. The results showed that building accurate, robust, and interpretable models for predicting the affinity data is totally possible, provided that suitable representations for proteins and ligands are used. Moreover, a further analysis showed that the model quality greatly depended on the sequence homology of proteins, and the model was very predictive only for proteins that had similar counterparts remaining in the model [72].

The main advantages of our proposed webserver are summarized as follows: (1) BioTriangle contains a selection of molecular features to analyze, classify, and compare complex molecular objects. They facilitate the exploitation of machine learning techniques to drive hypothesis from complex protein/peptide datasets, DNAs/RNAs datasets, small molecule datasets, and interaction datasets. (2) BioTriangle provides the detailed information about molecular descriptors and how to calculate them in the documentation section. This helps the researcher to understand the meaning of each descriptor and to interpret the model. (3) BioTriangle provides several tutorials and corresponding model scripts for different applications. This helps the researchers to apply BioTriangle into their data analysis pipeline for molecular representation. (4) BioTriangle provides various python scripts to several popular databases such as KEGG, PubChem, Drugbank, CAS, Uniprot, PDB, and GeneBank, etc., greatly facilitating the accessibility of molecular structures and sequences. (5) BioTriangle provides users online services, which means the tedious deployment or programming process of other tools mentioned above are no more needed. This would be very helpful for some pharmacologists and biological scientists with no programming skills. (6) The JavaScript and jQuery instead of Java applets are utilized to accomplish some complex interaction processes in the front-side of the server, which could effectively avoid potential problems of some strict runtime environment and security risks of Java.

The BioTriangle implementation of each of these algorithms was extensively tested by using a number of test proteins, DNAs/RNAs and small molecules. The computed descriptor values were also compared to the known values for these molecules from different software tools to ensure that their computation is accurate. For small molecular descriptors, we compared our calculated descriptors with those from Dragon, MOE (Molecular Operating Environment from Chemical Computing Group) or MODEL (Molecular Descriptor Lab). If our calculated descriptor is identical to those from these tools, we will confirm that this descriptor is correctly coded. For protein descriptors, we compared our calculated descriptors with those from PROFEAT (Protein Feature Server) or PseAAC server (http://www.csbio.sjtu.edu.cn/bioinf/PseAAC/). Similarly, if our calculated descriptor is identical to those from PROFEAT and PseAAC, we will conform that this protein descriptor is correctly calculated. For DNA/RNA descriptors, we compared our calculated descriptors with those from repDNA and repRNA (http://bioinformatics.hitsz.edu.cn/repRNA/), the identical results from two different tools demonstrated the accuracy of our calculated descriptors.

## Conclusion

BioTriangle provides a user-friendly interface to calculate various features of biological molecules and complex interaction samples conveniently. It makes a step in this direction providing a way to fully integrate information from chemical space and biology space into an interaction space, which cannot be performed by other existing web-based tools. It provides not only the detailed information about all descriptors and how to calculate them but also several tutorials and corresponding model scripts for different applications. In addition, the algorithms related in BioTriangle and the stability of the platform were extensively tested. We hope that the web service will be helpful when exploring questions concerning structures, functions and interactions of various molecular data in the context of computational biology. In future work, we plan to apply the integrated features on various biological research questions, and to extend the range of functions with new promising descriptors for the coming versions of BioTriangle.

## Availability and requirements

Project name: BioTriangle.

Project home page: http://biotriangle.scbdd.com.

Operating system(s): Platform independent.

Programming language: Python, JavaScript, HTML, CSS.

Other requirements: Modern internet browser supporting HTML5 and JavaScript. The recommended browsers: Safari, Firefox, Chrome, IE (Ver. >8).

License: http://creativecommons.org/licenses/by-nc-sa/4.0/.

Any restrictions to use by non-academics: License needed.

## Additional files

10.1186/s13321-016-0146-2 BioChem features.
10.1186/s13321-016-0146-2 BioProt features.
10.1186/s13321-016-0146-2 BioDNA features.
10.1186/s13321-016-0146-2 Features of BioCCI, BioPPI and BioDDI.
10.1186/s13321-016-0146-2 Features of BioCPI, BioDPI and BioCDI.
