# Supplementary material for: BioTriangle: a web-accessible platform for generating various molecular representations for chemicals, proteins, DNAs/RNAs and their interactions
Source: J Cheminform. 2016 Jun 21;8:34. doi: 10.1186/s13321-016-0146-2 (PMC4915156; doi:10.1186/s13321-016-0146-2)
Supplement: Supplementary file 2 — 10.1186/s13321-016-0146-2 BioProt features. [file 13321_2016_146_MOESM2_ESM.pdf]

BioTriangle

--BioProt features

COMPUTATIONAL BIOLOGY &
DRUG DESIGN GROUP
CENTRAL SOUTH UNIV., CHINA

Table of Contents

1 Features of protein and peptide 3

1.1 Amino acid composition 3

1.2 Dipeptide composition 3

1.3 Tripeptide composition 3

1.4 Autocorrelation descriptors 4

1.4.1 Normalized Moreau-Broto autocorrelation descriptors 4

1.4.2 Moran autocorrelation 5

1.4.3 Geary autocorrelation Descriptors 5

1.5 Composition, transition and distribution 6

1.6 Conjoint Triad Descriptors 8

1.7 Quasi-sequence-order Descriptors 10

1.7.1 Sequence-order-coupling numbers 10

1.7.2 Quasi-sequence-order (QSO) descriptors 11

1.8 pseudo-amino acid composition (PAAC) 12

1.9 Amphiphilic pseudo-amino acid composition (APAAC) 14

References: 16

1.10 Features list 18

©2015 Computational Biology&Drug Design Group, CSU, China

1 Features of protein and peptide

A protein or peptide sequence with N amino acid residues is expressed as: R_1, R_2, R_3, …, R_N, where R_i represents the residue at the i-th position in the sequence. The labels i and j are used to index amino acid position in a sequence and r, s are used to index the amino acid type. The computed features are divided into 4 groups according to their known applications described in the literature.

A protein sequence can be divided equally into segments and the methods, described as follows for the global sequence, can be applied to each segment.

1.1 Amino acid composition

The amino acid composition is the fraction of each amino acid type within a protein. The fractions of all 20 natural amino acids are calculated as:

$$f(r) = \frac{N_r}{N} \qquad r=1,2,3, ..., 20$$

Where N_r is the number of the amino acid type r and N is the length of the sequence.

1.2 Dipeptide composition

The dipeptide composition gives 400 features, defined as:

$$f(r,s) = \frac{N_{rs}}{N-1} \qquad r, s=1,2,3, ..., 20$$

where N_{rs} is the number of dipeptide represented by amino acid type r and s.

1.3 Tripeptide composition

The tripeptide composition gives 8000 features, defined as:

$$f(r,s,t) = \frac{N_{rst}}{N-2} \qquad r, s=1,2,3, ..., 20$$

where N_{rst} is the number of tripeptide represented by amino acid type r, s and t.

©2015 Computational Biology & Drug Design Group, CSU, China

1.4 Autocorrelation descriptors

Autocorrelation descriptors are defined based on the distribution of amino acid properties along the sequence. The amino acid properties used here are various types of amino acids index (http://www.genome.ad.jp/dbget/aaindex.html).Three type of autocorrelation descriptors are used here and are described as following.

All the amino acid indices are centralized and standardized before the calculation, i.e.

$$P_r = \frac{P_r - \overline{P}}{\sigma}$$

Where $\overline{P}$ is the average of the property of the 20 amino acids.

$$\overline{P} = \frac{\sum_{r=1}^{20} P_r}{20} \quad \text{and} \quad \sigma = \sqrt{\frac{1}{20}\sum_{r=1}^{20}(P_r - \overline{P})^2}$$

1.4.1 Normalized Moreau-Broto autocorrelation descriptors

Moreau-Broto autocorrelation descriptors application to protein sequences may be defined as:

$$AC(d) = \sum_{i=1}^{N-d} P_i P_{i+d} \qquad d=1,2,3,\ ...,\ \text{nlag}$$

Where d is called the lag of the autocorrelation and P_i and P_{i+d} are the properties of the amino acids at position i and $i+d$, respectively. *nlag* is the maximum value of the lag.

The normalized Moreau-Broto autocorrelation descriptors are defined as:

$$ATS(d) = \frac{AC(d)}{N-d} \qquad d=1,2,3,\ ...,\ nlag$$

©2015 Computational Biology& Drug Design Group, CSU, China

```
Database: AAindex
Entry: ANDN920101
LinkDB: ANDN920101

H ANDN920101
D alpha-CH chemical shifts (Andersen et al., 1992)
R LIT:1810048b PMID:1575719
A Andersen, N.H., Cao, B. and Chen, C.
T Peptide/protein structure analysis using the chemical shift index method:
  upfield alpha-CH values reveal dynamic helices and aL sites
J Biochem. and Biophys. Res. Comm. 184, 1008-1014 (1992)
C BUNA790102    0.949
I    A/L     R/K     N/M     D/F     C/P     Q/S     E/T     G/W     H/Y     I/V
    4.35    4.38    4.75    4.76    4.65    4.37    4.29    3.97    4.63    3.95
    4.17    4.36    4.52    4.66    4.44    4.50    4.35    4.70    4.60    3.95
//
```

Figure 2 An illustrated example in the AAIndex database

1.4.2 Moran autocorrelation

Moran autocorrelation descriptors application to protein sequence may be defined as:

$$I(d)=\frac{\frac{1}{N-d}\sum_{i=1}^{N-d}(P_i-\overline{P})(P_{i+d}-\overline{P})}{\frac{1}{N}\sum_{i=1}^{N}(P_i-\overline{P})^2}\qquad d=1,2,3,\ldots,30.$$

Where d and P_i and P_{i+d} are defined in the same way as in 2.2.1, and is the average of the considered property P along the sequence, i.e.,

$$\overline{P}=\frac{\sum_{i=1}^{N}P_i}{N}$$

Where d, $\overline{P}$, P_i and P_{i+d}, *nlag* have the same meaning as in the above.

1.4.3 Geary autocorrelation Descriptors

Geary autocorrelation descriptors application to protein sequence may be defined as:

©2015 Computational Biology & Drug Design Group, CSU, China

$$C(d)=\frac{\frac{1}{2(N-d)}\sum_{i=1}^{N-d}(P_i-P_{i+d})^2}{\frac{1}{N-1}\sum_{i=1}^{N}(P_i-\overline{P})^2}\qquad d=1,2,3,\ldots,30.$$

Where d, $\overline{P}$, P_i and P_{i+d} , *nlag* have the same meaning as in the above.

The amino acid indices used in these autocorrelation descriptors can be specified in file "input-param.dat" from "input-aaindexdb.dat".

For each amino acid index, there will be $3\times nlag$ autocorrelation descriptors.

1.5 Composition, transition and distribution

These descriptors are developed by Dubchak, et.al.

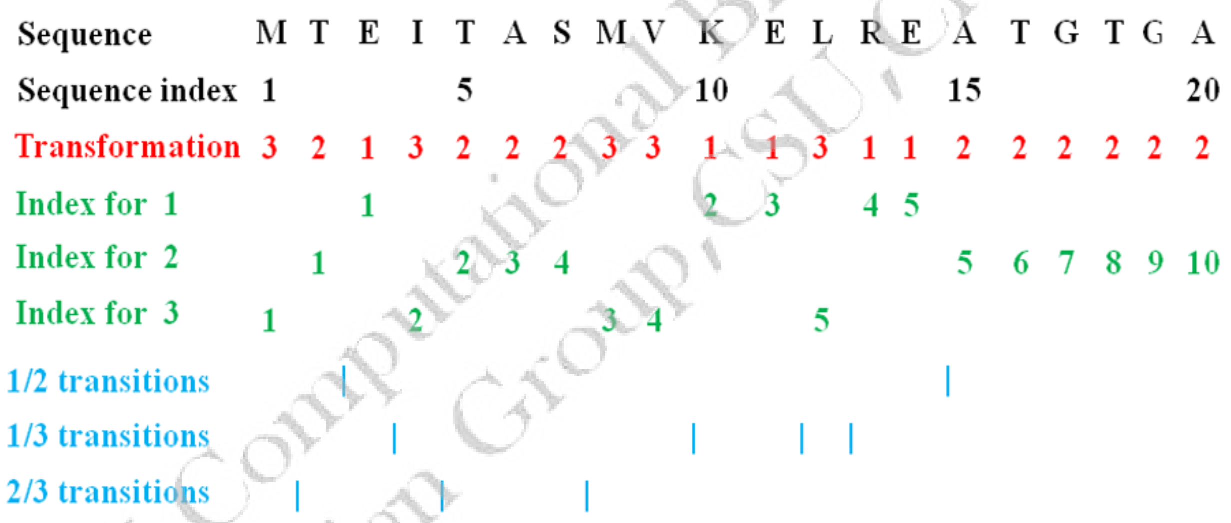

Figure 3 The sequence of a hypothetic protein indicating the construction of composition, transition and distribution descriptors of a protein. Sequence index indicates the position of an amino acid in the sequence. The index for each type of amino acids in the sequence ('1' '2' or '3') indicates the position of the first, second, third, ... of that type of amino acid. 1/2 transition indicates the position of '12' or '21' pairs in the sequence (1/3 and 2/3 are defined in the same way.).

Step1. Sequence encoding

The amino acids are divided in three classes according to its attribute and each amino acid is encoded by one of the indices 1, 2, 3 according to which class it belonged. The attributes used here include hydrophobicity, normalized van der Waals volume polarity, and polarizability, as in the references. The

©2015 Computational Biology & Drug Design Group, CSU, China

corresponding division is in the table 1.

Table 1 Amino acid attributes and the division of the amino acids into three groups for each attribute

	**Group 1**	**Group 2**	**Group 3**
hydrophobicity	Polar<br>R,K,E,D,Q,N	Neutral<br>G, A, S,T,P,H,Y	Hydrophobicity<br>C,L,V,I,M,F,W
normalized van der Waals volume	0-2.78<br>G,A,S,T,P,D	2.95-4.0<br>N,V,E,Q,I,L	4.03-8.08<br>M,H,K,F,R,Y,W
polarity	4.9-6.2<br>L,I,F,W,C,M,V,Y	8.0-9.2<br>P,A,T,G,S	10.4-13.0<br>H,Q,R,K,N,E,D
polarizability	0-1.08<br>G,A,S,D,T	0.128-0.186<br>C,P,N,V,E,Q,I,L	0.219-0.409<br>K,M,H,F,R,Y,W
charge	Positive<br>K,R	Neutral<br>A,N,C,Q,G,H,I,L,M,F,P,S,T,W,Y,V	Negative<br>D,E
secondary structure	Helix<br>E,A,L,M,Q,K,R,H	Strand<br>V,I,Y,C,W,F,T	Coil<br>G,N,P,S,D
solvent accessibility	Buried<br>A,L,F,C,G,I,V,W	Exposed<br>R,K,Q,E,N,D	Intermediate<br>M,S P,T,H,Y

For example, for a given sequence "MTEITAAMVKELRESTGAGA", it will be encoded as "32132223311311222222" according to its hydrophobicity division.

Step 2: Composition, Transition and Distribution descriptors

Three descriptors, "Composition (C)", "Transition (T)", and "Distribution (D)" were calculated for a given attribute as follows:

Composition: It is the global percent for each encoded class in the sequence. In the above example using hydrophobicity division, the numbers for encoded classes "1", "2", "3" are 5, 10, 5 respectively, so the compositions for them are 5/20=25%, 10/20=10%, and 5/20=25% respectively, where 20 is the length of the protein sequence. Composition can be defined as:

$$C_r = \frac{n_r}{n} \qquad r=1,2,3.$$

Where n_r is the number of r in the encoded sequence and N is the length of the sequence.

Transition: A transition from class 1 to 2 is the percent frequency with which 1 is followed by 2 or 2 is

©2015 Computational Biology&Drug Design Group, CSU, China

followed by 1 in the encoded sequence. Transition descriptor can be calculated as:

$$T_{rs} = \frac{n_{rs} + n_{sr}}{N - 1} \qquad rs="12", "13", "23".$$

Where n_{rs}, n_{sr} is the numbers of dipeptide encoded as "*rs*" and "*sr*" respectively in the sequence and N is the length of the sequence.

Distribution: The "distribution" descriptor describes the distribution of each attribute in the sequence. There are five "distribution" descriptors for each attribute and they are the position percents in the whole sequence for the first residue, 25% residues, 50% residues, 75% residues and 100% residues , respectively, for a specified encoded class. For example, there are10 residues encoded as "2" in the above example, the positions for the first residue "2", the 2th residue "2" (25%*10=2), the 5th "2" residue (50%*10=5), the 7th "2" (75%*10=7) and the10th residue "2" (100%*10) in the encoded sequence are 2, 5, 15, 17, 20 respectively, so the distribution descriptors for "2" are: 10.0 (2/20*100), 25.0 (5/20*100), 75.0 (15/20*100), 85.0 (17/20*100) , 100.0 (20/20*100), respectively.

1.6 Conjoint Triad Descriptors

Conjoint triad descriptors are proposed by J.W. Shen et.al. These conjoint triad features abstracts the features of protein pairs based on the classification of amino acids. In this approach, each protein sequence is represented by a vector space consisting of features of amino acids. To reduce the dimensions of vector space, the 20 amino acids were clustered into several classes according to their dipoles and volumes of the side chains. The conjoint triad features are calculated as follows:

Step 1: classification of amino acids

Electrostatic and hydrophobic interactions dominate protein-protein interactions. These two kinds of interactions may be reflected by the dipoles and volumes of the side chains of amino acids, respectively. Accordingly, these two parameters were calculated, respectively, by using the density-functional theory method B3LYP/6-31G and molecular modeling approach. Based on the dipoles and volumes of the side chains, the 20 amino acids could be clustered into seven classes (See Table 2). Amino acids within the same class likely involve synonymous mutations because of their similar characteristics.

©2015 Computational Biology & Drug Design Group, CSU, China

Table 2 Classification of amino acids based on dipoles and volumes of the side chains

No.	Dipole scale[a]	Volume scale[b]	Class
1	-	-	Ala, Gly, Val
2	-	+	Ile, Leu, Phe, Pro
3	+	+	Tyr, Met, Thr, Ser
4	++	+	His, Asn, Gln, Tpr
5	+++	+	Arg, Lys
6	+'+'+'	+	Asp, Glu
7	+ [c]	+	Cys

[a] Dipole scale (Debye): -, Dipole<1.0; +, 1.0<Dipole<2.0; ++, 2.0<Dipole<3.0; +++, Dipole>3.0; +'+'+', Dipole>3.0 with opposite orientation. [b] Volume scale (Å^3): -, Volume<50; +, Volume> 50. [c] Cys is separated from class 3 because of its ability to form disulfide bonds.

Step 2: Conjoint triad calculation

The conjoint triad descriptors considered the properties of one amino acid and its vicinal amino acids and regarded any three continuous amino acids as a unit. Thus, the triads can be differentiated according to the classes of amino acids, i.e., triads composed by three amino acids belonging to the same classes, such as ART and VKS, could be treated identically. To conveniently represent a protein, we first use a binary space (**V**, **F**) to represent a protein sequence. Here, **V** is the vector space of the sequence features, and each feature v_i represents a sort of triad type; **F** is the frequency vector corresponding to **V**, and the value of the *i*th dimension of **F** (f_i) is the frequency of type v_i appearing in the protein sequence. For the amino acids that have been catalogued into seven classes, the size of **V** should be 7×7×7; thus i = 1,2, ..., 343. The detailed description for (**V**, **F**) is illustrated in Figure 3. Clearly, each protein correlates to the length (number of amino acids) of protein. In general, a long protein would have a large value of f_i, which complicates the comparison between two heterogeneous proteins. Thus, we defined a new parameter, d_i, by normalizing f_i with the following equation.

$$d_i = (f_i - \min\{f_1, f_2, f_3, ..., f_{343}\}) / \max\{f_1, f_2, f_3, ..., f_{343}\}$$

The numerical value of d_i of each protein ranges from 0 to 1, which thereby enables the comparison between proteins. Accordingly, we obtain another vector space (designated **D**) consisting of d_i to represent protein

©2015 Computational Biology&Drug Design Group, CSU, China

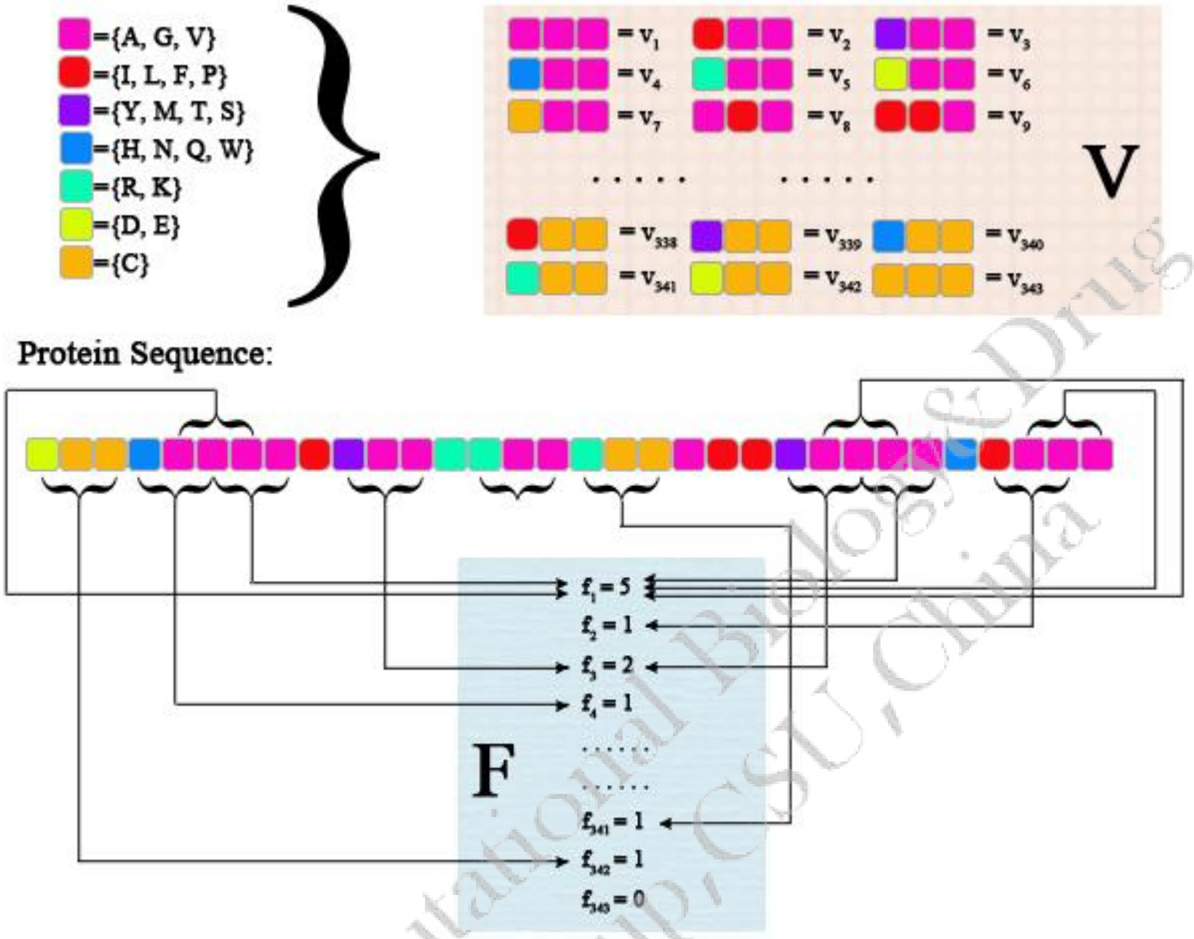

Figure 3 Schematic diagram for constructing the vector space (V, F) of protein sequence. V is the vector space of the sequence features; each feature (v_i) represents a triad composed of three consecutive amino acids; F is the frequency vector corresponding to V, and the value of the *i*th dimension of F(f_i) is the frequency that $\mathbf{v}_i$ triad appeared in the protein sequence.

1.7 Quasi-sequence-order Descriptors

The quasi-sequence-order descriptors are proposed by K.C. Chou, et.al. They are derived from the distance matrix between the 20 amino acids.

1.7.1 Sequence-order-coupling numbers

The dth-rank sequence-order-coupling number is defined as:

©2015 Computational Biology & Drug Design Group, CSU, China

$$\tau_d = \sum_{i=1}^{N-d} (d_{i,i+d})^2 \qquad d=1,2,3,\ ...,\ maxlag$$

Where $d_{i,i+d}$ is the distance between the two amino acids at position i and $i+d$.

Note: *maxlag* is the maximum lag and the length of the protein must be not less than *maxlag*.

1.7.2 Quasi-sequence-order (QSO) descriptors

For each amino acid type, a quasi-sequence-order descriptor can be defined as:

$$X_r = \frac{f_r}{\sum_{r=1}^{20} f_r + w \sum_{d=1}^{maxlag} \tau_d} \qquad r=1,2,3,\ ...,\ 20$$

Where f_r is the normalized occurrence for amino acid type i and w is a weighting factor (w=0.1). These are the first 20 quasi-sequence-order descriptors. The other 30 quasi-sequence-order are defined as:

$$X_r = \frac{w\tau_{d-20}}{\sum_{r=1}^{20} f_r + w \sum_{d=1}^{maxlag} \tau_d} \qquad d=21,22,23,\ ...,\ 20+maxlag$$

In addition to Schneider-Wrede physicochemical distance matrix used by Chou et al, another chemical distance matrix by Grantham is also used here.

©2015 Computational Biology&Drug Design Group, CSU, China

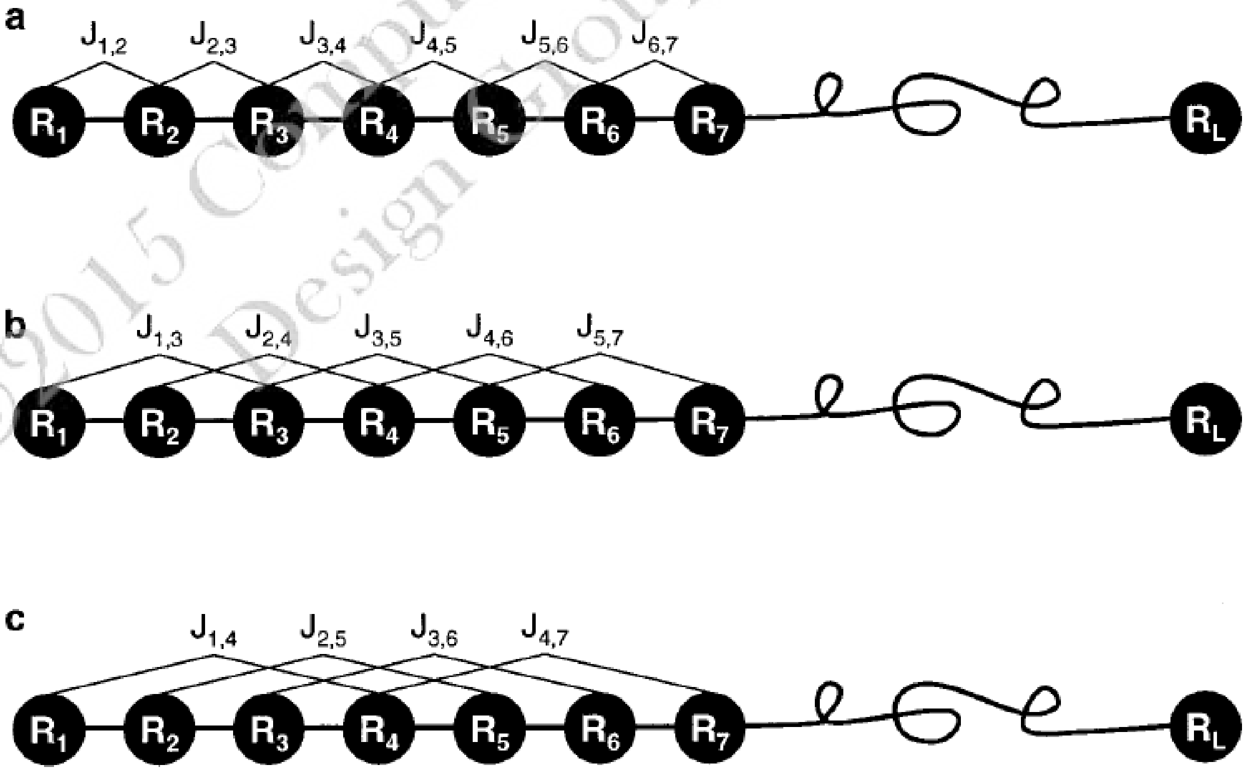

Figure 4 A schematic drawing to show (a) the 1st-rank, (b) the 2nd-rank, and (3) the 3rd-rank sequence-order-coupling mode along a protein sequence. (a) Reflects the coupling mode between all the most contiguous residues, (b) that between all the 2nd most contiguous residues, and (c) that between all the 3rd most contiguous residues.

1.8 pseudo-amino acid composition (PAAC)

This groups of descriptors are proposed by Kuo-chen Chou. PAAC descriptors (http://www.csbio.sjtu.edu.cn/bioinf/PseAAC/type1.htm) are also called the type 1 pseudo-amino acid composition. Let $H_1^o(i), H_2^o(i), M^o(i)$ (i=1,2,3, ..., 20) be the original hydrophobicity values, the original hydrophilicity values and the original side chain masses of the 20 natural amino acids, respectively. They are converted to following qualities by a standard conversion:

$$H_1(i) = \frac{H_1^o(i) - \frac{1}{20}\sum_{i=1}^{20} H_1^o(i)}{\sqrt{\frac{\sum_{i=1}^{20}[H_1^o(i) - \frac{1}{20}\sum_{i=1}^{20} H_1^o(i)]^2}{20}}}$$

$H_2^o(i)$ and $M^o(i)$ are normalized as $H_2(i)$ and $M(i)$ in the same way.

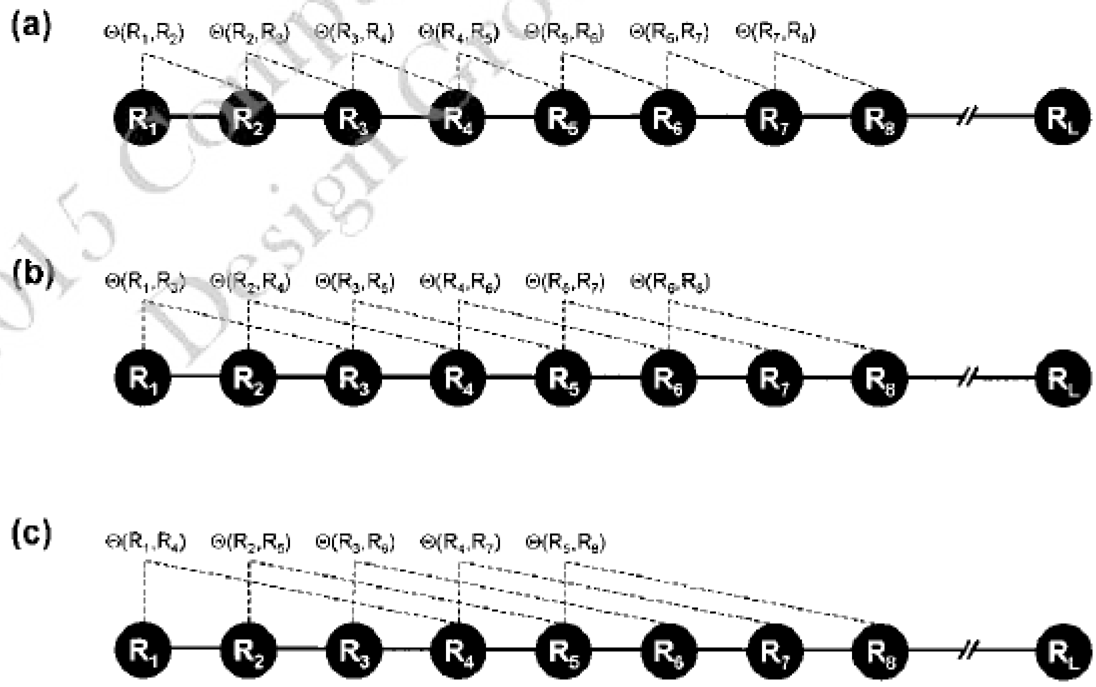

Figure 5 A schematic drawing to show (a) the first-tier, (b) the second-tier, and (3) the third-tier

©2015 Computational Biology & Drug Design Group, CSU, China

sequence order correlation mode along a protein sequence. Panel (**a**) reflects the correlation mode between all the most contiguous residues, panel (**b**) that between all the second-most contiguous residues, and panel (**c**) that between all the third-most contiguous residues.

Then, a correlation function can be defines as:

$$\Theta(R_i, R_j) = \frac{1}{3}\left\{\left[H_1(R_i) - H_1(R_j)\right]^2 + \left[H_2(R_i) - H_2(R_j)\right]^2 + \left[M(R_i) - M(R_j)\right]^2\right\}$$

This correlation function is actually an averaged value for the three amino acid properties: hydrophobicity value, hydrophilicity value and side chain mass. Therefore we can extend this definition of correlation function for one amino acid property or for a set of n amino acid properties. For one amino acid property, the correlation can be defined as:

$$\Theta(R_i, R_j) = \left[H_1(R_i) - H_1(R_j)\right]^2$$

where $H(R_i)$ is the amino acid property of amino acid R_i after standardization.

For a set of n amino acid properties, it can be defined as: where $H_k(R_i)$ is the kth property in the amino acid property set for amino acid R_i.

$$\Theta(R_i, R_j) = \frac{1}{n}\sum_{k=1}^{n}\left[H_k(R_i) - H_k(R_j)\right]^2$$

where Hk(Ri) is the kth property in the amino acid property set for amino acid Ri.

A set of descriptors called sequence order-correlated factors are defined as:

$$\theta_1 = \frac{1}{N-1}\sum_{i=1}^{N-1}\Theta(R_i, R_{i+1})$$

$$\theta_2 = \frac{1}{N-2}\sum_{i=1}^{N-2}\Theta(R_i, R_{i+2})$$

$$\theta_3 = \frac{1}{N-3}\sum_{i=1}^{N-3}\Theta(R_i, R_{i+3})$$

$$\ldots$$

$$\theta_\lambda = \frac{1}{N-\lambda}\sum_{i=1}^{N-\lambda}\Theta(R_i, R_{i+\lambda})$$

λ (<L) is a parameter to be chosen. Let f_i is the normalized occurrence frequency of the 20 amino acids in the protein sequence, a set of 20+λ descriptors called the pseudo-amino acid composition for a protein sequence can be defines as:

©2015 Computational Biology & Drug Design Group, CSU, China

$$Xc = \frac{f_c}{\sum_{r=1}^{20} f_r + w\sum_{j=1}^{\lambda}\theta_j} \qquad (1<c<20)$$

$$Xc = \frac{w\theta_{c-20}}{\sum_{r=1}^{20} f_r + w\sum_{j=1}^{\lambda}\theta_j} \qquad (21<c<20+\lambda)$$

where w is the weighting factor for the sequence-order effect and is set as w=0.05 in BioProt as suggested by Chou KC.

Note: the original hydrophobicity values for amino acids in BioProt are different from the values by Chou KC. In this updated version, the default values of amino acid properties are the values of Chou KC. However, in the work of Chou KC, the definition for "normalized occurrence frequency" is not given and in this work we define it as the occurrence frequency of amino acid in the sequence normalized to 100% and hence our calculated values are not the same as values by them.

1.9 Amphiphilic pseudo-amino acid composition (APAAC)

APAAC (http://www.csbio.sjtu.edu.cn/bioinf/PseAAC/type2.htm) are also called type 2 pseudo-amino acid composition. The definitions of these qualities are similar to the above PAAC descriptors. From $H_1(i)$ and $H_2(j)$ defined in eq. 16 and eq. 17, the hydrophobicity and hydrophilicity correlation functions are defined respectively as:

$$H^1_{i,j} = H_1(i)H_1(j)$$

$$H^2_{i,j} = H_2(i)H_2(j)$$

From these qualities, sequence order factors can be defines as:

$$\tau_1 = \frac{1}{N-1}\sum_{i=1}^{N-1} H^1_{i,i+1}$$

$$\tau_2 = \frac{1}{N-1}\sum_{i=1}^{N-1} H^2_{i,i+1}$$

$$\tau_3 = \frac{1}{N-2}\sum_{i=1}^{N-2} H^1_{i,i+2}$$

$$\tau_4 = \frac{1}{N-2}\sum_{i=1}^{N-2} H^2_{i,i+2}$$

...

©2015 Computational Biology & Drug Design Group, CSU, China

$$\tau_{2\lambda-1} = \frac{1}{N-\lambda}\sum_{i=1}^{N-\lambda} H^1_{i,i+\lambda}$$

$$\tau_{2\lambda} = \frac{1}{N-\lambda}\sum_{i=1}^{N-\lambda} H^2_{i,i+\lambda}$$

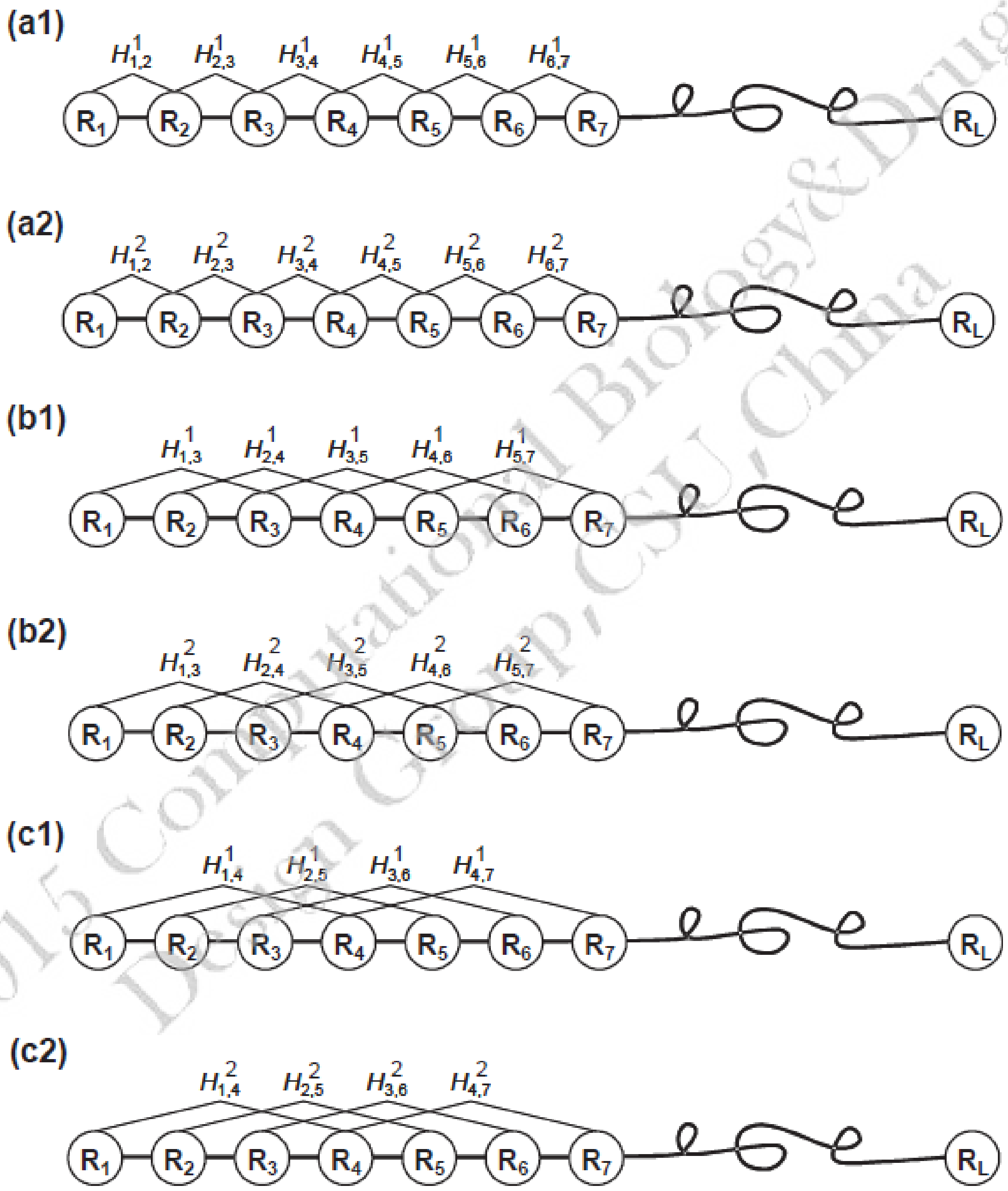

©2015 Computational Biology& Drug Design Group, CSU, China

Figure 6 A schematic diagram to show (**a1**/**a2**) the first-rank, (**b1**/**b2**) the second-rank and (**c1**/**c2**) the third-rank sequence-order-coupling mode along a protein sequence through a hydrophobicity/hydrophilicity correlation function, where $H^1_{i,j}$ and $H^2_{i,j}$ are given by Equation (3).

Panel (a1/a2) reflects the coupling mode between all the most contiguous residues, panel (b1/b2) that between all the second-most contiguous residues and panel (c1/c2) that between all the third-most contiguous residues.

Then a set of descriptors called “Amphiphilic pseudo amino acid composition” (APAAC) are defined as:

$$Pc = \frac{f_c}{\sum_{r=1}^{20} f_r + w\sum_{j=1}^{2\lambda}\tau_j} \qquad 1<c<20$$

$$Pc = \frac{w\tau_u}{\sum_{r=1}^{20} f_r + w\sum_{j=1}^{2\lambda}\tau_j} \qquad 21<u<20+2\lambda$$

Where w is the weighting factor and is taken as w=0.5 in BioProt as in the work of Chou KC.

References:

[1] M. Bhasin and G. P. S. Raghava. Classification of Nuclear Receptors Based on Amino Acid Composition and Dipeptide Composition. J. Bio. Chem. 2004, 279, 23262.

[2] Inna Dubchak, Ilya Muchink, Stephen R. Holbrook and Sung-Hou Kim. Prediction of protein folding class using global description of amino acid sequence. Proc. Natl. Acad. Sci. USA, 1995, 92, 8700-8704.

[3] Inna Dubchak, Ilya Muchink, Christopher Mayor, Igor Dralyuk and Sung-Hou Kim. Recognition of a Protein Fold in the Context of the SCOP classification. Proteins: Structure, Function and Genetics,1999,35,401-407.

[4] Kuo-Chen Chou. Prediction of Protein Subcellar Locations by Incorporating Quasi-Sequence-Order Effect. Biochemical and Biophysical Research Communications 2000, 278, 477-483.

[5] Kuo-Chen Chou and Yu-Dong Cai. Prediction of Protein sub-cellular locations by GO-FunD-PseAA predictor, Biochemical and Biophysical Research Communications, 2004, 320, 1236-1239.

[6] Gisbert Schneider and Paul wrede. The Rational Design of Amino Acid Sequences by Artificial Neural Networks and Simulated Molecular Evolution: Do Novo Design of an Idealized Leader Cleavage Site. Biophys Journal, 1994, 66, 335-344.

[7] Grantham, R. Amino acid difference formula to help explain protein evolution. Science, 1974, 185,

©2015 Computational Biology&Drug Design Group,CSU,China

862-864

[8] Kuo-Chen Chou. Prediction of Protein Cellular Attributes Using Pseudo-Amino Acid Composition. PROTEINS: Structure, Function, and Genetics,2001,43:246–255.

[9] Jiri Damborsky. Quantitative structure–function and structure–stability relationships of purposely modified proteins. Protein Engineering ,1998,11, 21-30

[10] Hopp-Woods. Prediction of protein antigenic determinants from amino acid sequences. Proc. Natl. Acad. Sci. 1981, 78, 3824-3828.

[11] http://www.csbio.sjtu.edu.cn/bioinf/PseAAC/

[12] Kuo-Chen Chou. Using amphiphilic pseudo amino acid composition to predict enzyme subfamily classes. Bioinformatics, 2005,21,10-19.

[13] J.W. Shen, J. Zhang, X.M. Luo, W.L. Zhu, K.Q. Yu, K.X. Chen, Y.X. Li, H.L. Jiang. Predicting protein-protein interactions based only on sequences information. Proc. Natl. Acad. Sci. 007, 104, 4337-4341.

[14] Z.R. Li, H.H. Lin, Y. Han, L. Jiang, X. Chen, Y.Z. Chen. PROFEAT: a web server for computing structural and physicochemical features of proteins and peptides form amino acid sequence. Nucleic Acids Research. 2006, 34, 32-37.

[15] H.B. Rao, F. Zhu, G.B. Yang, Z.R. Li, Y.Z. Chen. Update of PROFEAT: a web server for computing structural and physicochemical features of proteins and peptides from amino acid sequence. Nucleic Acids Research. 2011, 39, 385-390.

[16] Kawashima, S., Ogata, H., and Kanehisa, M.; AAindex: amino acid index database. Nucleic Acids Res. 27, 368-369 (1999).

[17] Kawashima, S. and Kanehisa, M.; AAindex: amino acid index database. Nucleic Acids Res. 28, 374 (2000).

[18] Kawashima, S., Pokarowski, P., Pokarowska, M., Kolinski, A., Katayama, T., and Kanehisa, M.; AAindex: amino acid index database, progress report 2008. Nucleic Acids Res. 36, D202-D205 (2008).

©2015 Computational Biology& Drug Design Group, CSU, China

1.10 Features list

Table S1 List of BioProt computed features for protein sequences

Feature group	Features	Number of descriptors
Amino acid composition	Amino acid composition	20
	Dipeptide composition	400
	Tripeptide composition	8000
Autocorrelation	Normalized Moreau-Broto autocorrelation	240[a]
	Moran autocorrelation	240[a]
	Geary autocorrelation	240[a]
CTD	Composition	21
	Transition	21
	Distribution	105
Conjoint Triad	Conjoint Triad	343
Quasi-sequence order	Sequence order coupling number	60
	Quasi-sequence order descriptors	100
Pseudo amino acid composition	Pseudo amino acid composition	50[b]
	Amphiphilic pseudo amino acid composition	50[c]

[a] The number depends on the choice of the number of properties of amino acid and the choice of the maximum values of the *lag*. The default is use eight types of properties and *lag* = 30.

[b] The number depends on the choice of the number of the set of amino acid properties and the choice of the *lamda* value. The default is use three types of properties proposed by Chou et al and *lamda* = 30.

[c] The number depends on the choice of the *lamda* vlaue. The default is that *lamda* = 30.

©2015 Computational Biology& Drug Design Group, CSU, China

©2015 Computational Biology& Drug Design Group,CSU,China
