# Supplementary material for: BioTriangle: a web-accessible platform for generating various molecular representations for chemicals, proteins, DNAs/RNAs and their interactions
Source: J Cheminform. 2016 Jun 21;8:34. doi: 10.1186/s13321-016-0146-2 (PMC4915156; doi:10.1186/s13321-016-0146-2)
Supplement: Supplementary file 3 — 10.1186/s13321-016-0146-2 BioDNA features. [file 13321_2016_146_MOESM3_ESM.pdf]

BioTriangle

--BioDNA features

COMPUTATIONAL BIOLOGY &
DRUG DESIGN GROUP
CENTRAL SOUTH UNIV., CHINA

Table of contents

1. Nucleic acid composition..3

1.1 Basic kmer 3

1.2 Reverse compliment kmer 3

1.3 Increment of diversity...4

2. Autocorrelation 4

2.1 Dinucleotide-based auto covariance 5

2.2 Dinucleotide-based cross covariance...6

2.3 Dinucleotide-based auto-cross covariance...6

2.4 Trinucleotide-based auto covariance...7

2.5 Trinucleotide-based cross covariance 7

2.6 Trinucleotide-based auto-cross covariance 8

3. Pseudo nucleic acid composition...9

3.1 Pseudo dinucleotide composition 9

3.2 Pseudo *k*-tupler composition...10

3.3 Parallel correlation pseudo dinucleotide composition 11

3.4 Parallel correlation pseudo trinucleotide composition..................................13

3.5 Series correlation pseudo dinucleotide composition.....................................14

3.6 Series correlation pseudo trinucleotide composition 15

4. Table 1..17

5.Table 2...17

6.Table 3...18

©2015 Computational Biology & Drug Design Group, CSU, China

1. Nucleic acid composition

The most straight forward approach to represent the DNA sequences is based on their nucleic acid composition. The kmer and its variants have been widely used for this aim. Here, BioDNA allows users to calculate various kinds of kmer-based feature vectors for given sequences or FASTA files by selecting different methods and parameters. This module aims at computing three types of nucleic acid composition, including basic kmer, reverse compliment kmer and increment of diversity. Let's introduce them one by one.

1.1 Basic kmer

Basic kmer is the simplest approach to represent the DNAs, in which the DNA sequences are represented as the occurrence frequencies of k neighboring nucleic acids. This approach has been successfully applied to human gene regulatory sequence prediction (Noble, et al., 2005), enhancer identification (Lee, et al., 2011), etc.

The parameters:

- *k*: the *k* value of kmer, it should be an integer larger than 0.
- *normalize*: with this option, the final feature vector will be normalized based on the total occurrences of all kmers. Therefore, the elements in the feature vectors represent the frequencies of kmers. The default value of this parameter is **True**.

©2015 Computational Biology&Drug Design Group, CSU, China

1.2 Reverse compliment kmer

The reverse compliment kmer is a variant of the basic kmer, in which the kmers are not expected to be strand-specific, so reverse complements are collapsed into a single feature. For example, if k=2, there are totally 16 basic kmers ('AA', 'AC', 'AG', 'AT', 'CA', 'CC', 'CG', 'CT', 'GA', 'GC', 'GG', 'GT', 'TA', 'TC', 'TG', 'TT'), but by removing the reverse compliment kmers, there are only 10 distinct kmers in the reverse compliment kmer approach ('AA', 'AC', 'AG', 'AT', 'CA', 'CC', 'CG', 'GA', 'GC', 'TA'). For more information of this approach, please refer to (Noble, et al., 2005) (Gupta, et al., 2008)

The parameters:

- *k*: the *k* value of kmer, it should be an integer larger than 0.
- *normalize*: with this option, the final feature vector will be normalized based on the total occurrences of all kmers. Therefore, the elements in the feature vector represent the frequencies of kmers. The default value of this parameter is **True**.

1.3 Increment of diversity

The increment of diversity has been successfully applied in the prediction of exon-intron splice sites for several model genomes (Zhang and Luo, 2003), transcription start site prediction, and studying the organization of nucleosomes around splice sites(Lv and Luo, 2008).

In this method, the sequence features are converted into the increment of diversity (ID), defined by the relation of sequence X with standard source S:

$$ID = Diversity(X+S) - Diversity(S) - Diversity(X) \qquad (2)$$

Given a sequence X with r feature variables (ID_1 to ID_r), we obtain an r-dimensional feature vector $\boldsymbol{R}$ = (ID_1 , ID_2 , …, ID_r). The feature vector $\boldsymbol{R}$ is designed by the following considerations. The kmers are responsible for the discrimination between positive samples and negative samples, and therefore they construct the diversity sources. Based on this, 2 kmer-based increments of diversities ID_1 (ID_2) between sequence X and the standard source in positive (negative) training set can be easily introduced as the feature vectors.

For more information of this approach, please refer to (Chen, et al., 2010) and (Liu, et al., 2012).

The parameters:
- *k*: the *k* value of kmer, it should be an integer larger than 0, the default value is 6.

Note: This feature is temporally not included in BioDNA (Version 1.0).

2. Autocorrelation

Autocorrelation, as one of the multivariate modeling tools, can transform the DNA sequences of different lengths into fixed-length vectors by measuring the correlation between any two properties. Autocorrelation results in two kinds of variables: autocorrelation (AC) between the same property, and cross-covariance (CC) between

©2015 Computational Biology&Drug Design Group, CSU China

two different properties. Here, BioDNA allows users to calculate various kinds of autocorrelation feature vectors for given DNA sequences or FASTA files by selecting different methods and parameters. This module aims at computing six types of autocorrelation, including dinucleotide-based auto covariance (DAC), dinucleotide-based cross covariance (DCC), dinucleotide-based auto-cross covariance (DACC), trinucleotide-based auto covariance (TAC), trinucleotide-based cross covariance (TCC), and trinucleotide-based auto-cross covariance (TACC). Let's introduce them one by one.

2.1 Dinucleotide-based auto covariance

Suppose a DNA sequence D with L nucleic acid residues; i.e.

$$\mathbf{D} = \mathrm{R}_1\mathrm{R}_2\mathrm{R}_3\mathrm{R}_4\mathrm{R}_5\mathrm{R}_6\mathrm{R}_7 \cdots \mathrm{R}_L \quad (3)$$

where R_1 represents the nucleic acid residue at the sequence position 1, R_2 the nucleic acid residue at position 2 and so forth.

The DAC measures the correlation of the same physicochemical index between two dinucleotide separated by a distance of *lag* along the sequence, which can be calculated as:

$$\mathrm{DAC}(u, lag) = \sum_{i=1}^{L-lag-1} (P_u(\mathrm{R}_i\mathrm{R}_{i+1}) - \overline{P_u})(P_u(\mathrm{R}_{i+lag}\mathrm{R}_{i+lag+1}) - \overline{P_u}) / (L - lag - 1) \quad (4)$$

where u is a physicochemical index, L is the length of the DNA sequence, P_u (R_i *R_{i+1}) means the numerical value of the physicochemical index u for the dinucleotide R_i *R_{i+1} at position i, $\overline{P_u}$ is the average value for physicochemical index u along the whole sequence:

$$\overline{P_u} = \sum_{j=1}^{L-1} P_u(\mathrm{R}_j\mathrm{R}_{j+1}) / (L-1) \quad (5)$$

In such a way, the length of DAC feature vector is N∗LAG, where N is the number of physicochemical indices and LAG is the maximum of *lag* (*lag* = 1, 2, …, LAG).

This DAC approach is similar as the approach used for protein fold recognition (Dong, et al., 2009).

The parameters:
- *lag*: an integer larger than or equal to 0 and less than or equal to *L-2* (*L* means the length of the shortest DNA sequence in the dataset). It represents the distance between two dinucleotides.
- *phyche_index*: the physicochemical indices, it should be a list type and there are 38 different physicochemical indices (**Table 1**), which the users can choose.

©2015 Computational Biology&Drug Design Group, CSU, China

2.2 Dinucleotide-based cross covariance

Given a DNA sequence D (**Eq. 3**), the DCC approach measures the correlation of two different physicochemical indices between two dinucleotides separated by lag nucleic acids along the sequence, which can be calculated by:

$$\mathrm{DCC}(u_1,u_2,lag)=\sum_{i=1}^{L-lag-1}(P_{u_1}(R_iR_{i+1})-\overline{P}_{u_1})(P_{u_2}(R_{i+lag}R_{i+lag+1})-\overline{P}_{u_2})/(L-lag-1) \quad (6)$$

where u_1, u_2 are two different physicochemical indices, L is the length of the DNA sequence, $P_{u_1}(\mathrm{R}_i\mathrm{R}_{i+1})$ ($P_{u_2}(\mathrm{R}_i\mathrm{R}_{i+1})$) is the numerical value of the physicochemical index u_1 (u_2) for the dinucleotide R_i *R_{i+1} at position i, $\overline{P}_{u_1}$ ($\overline{P}_{u_2}$) is the average value for physicochemical index value u_1, u_2 along the whole sequence:

$$\overline{P}_u=\sum_{j=1}^{L-1}P_u(\mathrm{R}_j\mathrm{R}_{j+1})/(L-1) \quad (7)$$

In such a way, the length of the DCC feature vector is N*(N-1)*LAG, where N is the number of physicochemical indices and LAG is the maximum of *lag* (*lag*=1, 2, …, LAG).

This DCC approach is similar as the approach used for protein fold recognition (Dong, et al., 2009).

The parameters:

- *lag*: an integer larger than or equal to 0 and less than or equal to *L-2* (*L* means the length of the shortest DNA sequence in the dataset). It represents the distance between two dinucleotides.
- *phyche_index*: the physicochemical indices, it should be a list type and there are 38 different physicochemical indices (**Table 1**), which the users can choose.

©2015 Computational Biology & Drug Design Group, CSU, China

2.3 Dinucleotide-based auto-cross covariance

DACC is a combination of DAC and DCC. Therefore, the length of the DACC feature vector is N*N*LAG, where N is the number of physicochemical indices and LAG is the maximum of *lag* (*lag* = 1, 2, …, LAG).

The parameters:

- *lag*: an integer larger than or equal to 0 and less than or equal to *L-2* (*L* means the

length of the shortest DNA sequence in the dataset). It represents the distance between two dinucleotides.

- *phyche_index*: the physicochemical indices, it should be a list type and there are 38 different physicochemical indices (**Table 1**), which the users can choose.

2.4 Trinucleotide-based auto covariance

Given a DNA sequence D (**Eq. 3**), the TAC approach measures the correlation of the same physicochemical index between two trinucleotides separated by *lag* nucleic acids along the sequence, which can be calculated as:

$$\mathrm{TAC}(lag,u)=\sum_{i=1}^{L-lag-2}(P_u(\mathrm{R}_i\mathrm{R}_{i+1}\mathrm{R}_{i+2})-\overline{P_u})(P_u(\mathrm{R}_{i+lag}\mathrm{R}_{i+lag+1}\mathrm{R}_{i+lag+2})-\overline{P_u})/(L-lag-2) \tag{8}$$

where u is a physicochemical index, L is the length of the DNA sequence, $P_u(\mathrm{R}_i\mathrm{R}_{i+1}\mathrm{R}_{i+2})$ represents the numerical value of the physicochemical index u for the trinucleotide $\mathrm{R}_i\mathrm{R}_{i+1}\mathrm{R}_{i+2}$ at position i, $\overline{P_u}$ is the average value for physicochemical index u value along the whole sequence:

$$\overline{P_u}=\sum_{j=1}^{L-2}P_u(\mathrm{R}_j\mathrm{R}_{j+1}\mathrm{R}_{j+2})/(L-2) \tag{9}$$

In such a way, the length of TAC feature vector is $N*$LAG, where N is the number of physicochemical indices and LAG is the maximum of *lag* (*lag*=1, 2, …, LAG).

The parameters:

- *lag*: an integer larger than or equal to 0 and less than or equal to L-3 (L means the length of the shortest DNA sequence in the dataset). It represents the distance between two trinucleotides.
- *phyche_index*: the physicochemical indices, it should be a list and there are 12 different physicochemical indices (**Table 2**), which the users can choose.

2.5 Trinucleotide-based cross covariance

Given a DNA sequence D (**Eq. 3**), the TCC approach measures the correlation of two different physicochemical indices between two trinucleotides separated by *lag* nucleic acids along the sequence, which can be calculated by:

©2015 Computational Biology & Drug Design Group, CSU, China

$$\mathrm{TCC}(u_1,u_2,\mathrm{lag})=\sum_{i=1}^{L-lag-2}(P_{u_1}(\mathrm{R}_i\mathrm{R}_{i+1}\mathrm{R}_{i+2})-\overline{P}_{u_1})(P_{u_2}(\mathrm{R}_{i+lag}\mathrm{R}_{i+lag+1}\mathrm{R}_{i+lag+2})-\overline{P}_{u_2})/(L\text{-}lag\text{-}2) \quad (10)$$

where u_1, u_2 are two physicochemical indices, L is the length of the DNA sequence, $P_{u_1}(\mathrm{R}_i\mathrm{R}_{i+1}\mathrm{R}_{i+2})$ $(P_{u_2}(\mathrm{R}_i\mathrm{R}_{i+1}\mathrm{R}_{i+2}))$ represents the numerical value of the physicochemical index u_1 (u_2) for the trinucleotide $\mathrm{R}_i\mathrm{R}_{i+1}\ \mathrm{R}_{i+2}$ at position *i*, $\overline{P}_{u_1}$ $(\overline{P}_{u_2})$ is the average value for physicochemical index value u_1 (u_2) along the whole sequence:

$$\overline{P}_u=\sum_{j=1}^{L-2}P_u(\mathrm{R}_j\mathrm{R}_{j+1}\mathrm{R}_{j+2})/(L-2) \quad (11)$$

In such a way, the length of TCC feature vector is *N**(*N*-1)*LAG, where N is the number of physicochemical index and LAG is the maximum of *lag* (*lag* = 1, 2, …, LAG).

The parameters:

- *lag*: an integer larger than or equal to 0 and less than or equal to *L*-3 (*L* means the length of the shortest sequence in the dataset). It represents the distance between two trinucleotides.
- *Phyche_index*: the physicochemical indices, it should be a list and there are 12 different physicochemical indices (**Table 2**), which the users can choose.

2.6 Trinucleotide-based auto-cross covariance

TACC is a combination of TAC and TCC. Therefore, the length of the TACC feature vector is *N***N**LAG, where *N* is the number of physicochemical indices and LAG is the maximum of *lag* (*lag* = 1, 2, …, LAG).

The parameters:

- *lag*: an integer larger than or equal to 0 and less than or equal to L-3 (L means the length of the shortest DNA sequence in the dataset). It represents the distance between two trinucleotides.
- *phyche_index*: the physicochemical indices, it should be a list and there are 12 different physicochemical indices (**Table 2**), which the users can choose.

©2015 Computational Biology&Drug Design Group, CSU, China

3. Pseudo nucleic acid composition

PseNAC is a kind of powerful approaches to represent the DNA sequences considering both DNA local sequence-order information and long range or global sequence-order effects. Here, BioDNA allows users to calculate various kinds of PseNAC based feature vectors for given sequences or FASTA files by selecting different methods and parameters. This module aims at computing six types of pseudo nucleic acid composition: pseudo dinucleotide composition (PseDNC), pseudo k-tuple nucleotide composition (PseKNC), parallel correlation pseudo dinucleotide composition (PC-PseDNC), parallel correlation pseudo trinucleotide composition (PC-PseTNC), series correlation pseudo dinucleotide composition (SC-PseDNC), and series correlation pseudo trinucleotide composition (SC-PseTNC). Let's introduce them one by one.

3.1 Pseudo dinucleotide composition

PseDNC is an approach incorporating the contiguous local sequence-order information and the global sequence-order information into the feature vector of the DNA sequence.

Given a DNA sequence D (**Eq. 3**), the feature vector of D is defined:

$$\mathbf{D} = [d_1\ d_2\ \cdots\ d_{16}\ d_{16+1}\ \cdots\ d_{16+\lambda}]^{\mathrm{T}} \quad (12)$$

where

$$d_k = \begin{cases} \dfrac{f_k}{\sum_{i=1}^{16} f_i + w\sum_{j=1}^{\lambda}\theta_j} & (1 \le k \le 16) \\ \dfrac{w\theta_{k-16}}{\sum_{i=1}^{16} f_i + w\sum_{j=1}^{\lambda}\theta_j} & (17 \le k \le 16+\lambda) \end{cases} \quad (13)$$

where $f_k\,(k=1,2,\cdots,16)$ is the normalized occurrence frequency of dinucleotide in the DNA sequence; the parameter λ is an integer, representing the highest counted rank (or tier) of the correlation along a DNA sequence; *w* is the weight factor ranged from 0 to 1; $\theta_j\,(j=1,2,\cdots,\lambda)$ is called the j-tier correlation factor that reflects the sequenceorder correlation between all the most contiguous dinucleotide along a DNA sequence, which is defined:

©2015 Computational Biology & Drug Design Group, CSU, China

$$\begin{cases} \theta_1 = \frac{1}{L-2}\sum_{i=1}^{L-2}\Theta(R_iR_{i+1}, R_{i+1}R_{i+2}) \\ \theta_2 = \frac{1}{L-3}\sum_{i=1}^{L-3}\Theta(R_iR_{i+1}, R_{i+2}R_{i+3}) \\ \theta_3 = \frac{1}{L-4}\sum_{i=1}^{L-4}\Theta(R_iR_{i+1}, R_{i+3}R_{i+4}) \\ \quad \ldots\ldots \\ \theta_\lambda = \frac{1}{L-1-\lambda}\sum_{i=1}^{L-1-\lambda}\Theta(R_iR_{i+1}, R_{i+\lambda}R_{i+\lambda+1}) \end{cases} \quad (\lambda < L) \qquad (14)$$

$$\begin{cases} \theta_1 = \frac{1}{L-2}\sum_{i=1}^{L-2}\Theta(R_iR_{i+1}, R_{i+1}R_{i+2}) \\ \theta_2 = \frac{1}{L-3}\sum_{i=1}^{L-3}\Theta(R_iR_{i+1}, R_{i+2}R_{i+3}) \\ \theta_3 = \frac{1}{L-4}\sum_{i=1}^{L-4}\Theta(R_iR_{i+1}, R_{i+3}R_{i+4}) \\ \quad \ldots\ldots \\ \theta_\lambda = \frac{1}{L-1-\lambda}\sum_{i=1}^{L-1-\lambda}\Theta(R_iR_{i+1}, R_{i+\lambda}R_{i+\lambda+1}) \end{cases} \quad (\lambda < L) \qquad (14)$$

where the correlation function is given by

$$\Theta(R_iR_{i+1}, R_jR_{j+1}) = \frac{1}{\mu}\sum_{u=1}^{\mu}[P_u(R_iR_{i+1}) - P_u(R_jR_{j+1})]^2 \qquad (15)$$

where μ is the number of physicochemical indices, in this study, 6 indices reflecting the local DNA structural properties (Table 3) were employed to generate the PseDNC feature vector; $P_u(R_iR_{i+1})$ represents the numerical value of the u-th (u = 1, 2,… μ) physicochemical index of the dinucleotide R_iR_{i+1} at position i and $P_u(R_jR_{j+1})$ represents the corresponding value of the dinucleotide R_jR_{j+1} at position j. For more information about this approach, please refer to (Chen, et al., 2013).

The parameters:

- *lamada*: an integer larger than or equal to 0 and less than or equal to L-2 (L means the length of the shortest sequence in the dataset). It represents the highest counted rank (or tier) of the correlation along a DNA sequence. Its default value is 3.
- *w*: the weight factor ranged from 0 to 1. Its default value is 0.05.

3.2 Pseudo *k*-tupler composition

PseKNC improved the PseDNC approach by incorporating *k*-tuple nucleotide

©2015 Computational Biology & Drug Design Group CSU, China

composition.
Given a DNA sequence D (**Eq. 3**), the feature vector of D is defined:

$$\mathbf{D} = [d_1\ d_2 \cdots d_{4^k}\ d_{4^k+1} \cdots d_{4^k+\lambda}]^{\mathrm{T}} \tag{16}$$

where

$$d_u = \begin{cases} \dfrac{f_u}{\sum_{i=1}^{4^k} f_i + w\sum_{j=1}^{\lambda}\theta_j} (1 \le u \le 4^k) \\ \dfrac{w\theta_{u-4^k}}{\sum_{i=1}^{4^k} f_i + w\sum_{j=1}^{\lambda}\theta_j} (4^k \le u \le 4^k + \lambda) \end{cases} \tag{17}$$

where λ is the number of the total counted ranks (or tiers) of the correlations along a DNA sequence; f_u $(u=1,2,\cdots,4^k)$ is the frequency of oligonucleotide that is normalized to $\sum_{i=1}^{4^k} f_i = 1$; w is a weight factor; θ_j is given by

$$\theta_j = \frac{1}{L\text{-}j\text{-}1}\sum_{i=1}^{L-j-1}\Theta(\mathrm{R}_i\mathrm{R}_{i+1}, \mathrm{R}_{i+j}\mathrm{R}_{i+j+1}) (j = 1, 2, \cdots, \lambda; \lambda < L) \tag{18}$$

which represents the j-tier structural correlation factor between all the jth most contiguous dinucleotides. The correlation function $\Theta(\mathrm{R}_i\mathrm{R}_{i+1}, \mathrm{R}_{i+j}\mathrm{R}_{i+j+1})$ is defined by

$$\Theta(\mathrm{R}_i\mathrm{R}_{i+1}, \mathrm{R}_{i+j}\mathrm{R}_{i+j+1}) = \frac{1}{\mu}\sum_{v=1}^{\mu}[P_v(\mathrm{R}_i\mathrm{R}_{i+1}) - P_v(\mathrm{R}_{i+j}\mathrm{R}_{i+j+1})]^2 \tag{19}$$

where μ is the number of physicochemical indices, in this study, 6 indices reflecting the local DNA structural properties (**Table 3**) were employed to generate the PseKNC feature vector; $P_v(\mathrm{R}_i\mathrm{R}_{i+1})$ represents the numerical value of the v-th (u = 1, 2,… μ) physicochemical indices for the dinucleotide $\mathrm{R}_i\mathrm{R}_{i+1}$ at position i and $P_v(\mathrm{R}_{i+j}\mathrm{R}_{i+j+1})$ represents the corresponding value for the dinucleotide $\mathrm{R}_{i+j}\mathrm{R}_{i+j+1}$ at position i+j.
For more information about this approach, please refer to (Guo, et al., 2014)

The parameters:

- *k*: an integer larger than 0 represents the *k*-tuple. Its default value is 3.
- *lamada*: an integer larger than or equal to 0 and less than or equal to *L*-2 (*L* means the length of the shortest DNA sequence in the dataset), representing the highest counted rank (or tier) of the correlation along a DNA sequence. The default value is 1.
- *w*: the weight factor ranged from 0 to 1. Its default value is 0.05.

3.3 Parallel correlation pseudo dinucleotide composition

In PC-PseDNC approach, the users cannot only select the 38 built-in physiochemical

©2015 Computational Biology & Drug Design Group, CSU China

indices (**Table 1**), but also can upload their own indices to generate the PC-PseDNC feature vector.

Given a DNA sequence D (**Eq. 3**), the PC-PseDNC feature vector of **D** is defined:

$$\mathbf{D} = [d_1 \ d_2 \ \cdots \ d_{16} \ d_{16+1} \ \cdots \ d_{16+\lambda}]^{\mathrm{T}} \tag{20}$$

where

$$d_k = \begin{cases} \dfrac{f_k}{\sum_{i=1}^{16} f_i + w\sum_{j=1}^{\lambda} \theta_j} & (1 \le k \le 16) \\[2ex] \dfrac{w\theta_{k-16}}{\sum_{i=1}^{16} f_i + w\sum_{j=1}^{\lambda} \theta_j} & (17 \le k \le 16+\lambda) \end{cases} \tag{21}$$

where f_k（k=1,2,⋯,16) is the normalized occurrence frequency of dinucleotide in the DNA sequence; the parameter λ is an integer, representing the highest counted rank (or tier) of the correlation along a DNA sequence; w is the weight factor ranged from 0 to 1; θ_j (j=1, 2, ⋯, λ) is called the j-tier correlation factor that reflects the sequence order correlation between all the most contiguous dinucleotides along a DNA sequence, which is defined:

$$\begin{cases} \theta_1 = \dfrac{1}{L\text{-}2}\sum_{i=1}^{L-2} \Theta(\mathrm{R}_i\mathrm{R}_{i+1}, \mathrm{R}_{i+1}\mathrm{R}_{i+2}) \\ \theta_2 = \dfrac{1}{L\text{-}3}\sum_{i=1}^{L-3} \Theta(\mathrm{R}_i\mathrm{R}_{i+1}, \mathrm{R}_{i+2}\mathrm{R}_{i+3}) \\ \theta_3 = \dfrac{1}{L\text{-}4}\sum_{i=1}^{L-4} \Theta(\mathrm{R}_i\mathrm{R}_{i+1}, \mathrm{R}_{i+3}\mathrm{R}_{i+4}) \qquad (\lambda < L) \\ \quad \cdots\cdots \\ \theta_\lambda = \dfrac{1}{L\text{-}1\text{-}\lambda}\sum_{i=1}^{L-1-\lambda} \Theta(\mathrm{R}_i\mathrm{R}_{i+1}, \mathrm{R}_{i+\lambda}\mathrm{R}_{i+\lambda+1}) \end{cases} \tag{22}$$

where the correlation function is given by

$$\Theta(\mathrm{R}_i\mathrm{R}_{i+1}, \mathrm{R}_j\mathrm{R}_{j+1}) = \frac{1}{\mu}\sum_{u=1}^{\mu}[P_u(\mathrm{R}_i\mathrm{R}_{i+1}) - P_u(\mathrm{R}_j\mathrm{R}_{j+1})]^2 \tag{23}$$

where μ is the number of physicochemical indices considered that are listed in the **Table 1**; $P_u(\mathrm{R}_i\mathrm{R}_{i+1})$ ($P_u(\mathrm{R}_j\mathrm{R}_{j+1})$) represents the numerical value of the u-th (u = 1, 2,… μ) physicochemical index for the dinucleotide $\mathrm{R}_i\mathrm{R}_{i+1}$ ($\mathrm{R}_j\mathrm{R}_{j+1}$) at position i and j, respectively.

For more information of PC-PseDNC approach, you can refer to (Chen, et al., 2014).

The parameters:

- *lamada*: an integer larger than or equal to 0 and less than or equal to L-2 (L means the length of the shortest DNA sequence in the dataset), representing the highest counted rank (or tier) of the correlation along a DNA sequence. Its default value is 1.
- *w*: the weight factor ranged from 0 to 1, its default value is 0.05.

©2015 Computational Biology & Drug Design Group, CSU, China

- *phyche_index*: The 38 built-in physicochemical indices (**Table 1**), which the users can choose. Its type should be a list.

3.4 Parallel correlation pseudo trinucleotide composition

In PC-PseTNC approach, 12 built-in trinucleotide physiochemical indices (**Table 2**) are incorporated to generate the representations of DNA sequences. Furthermore, the user defined indices can be also used to generate the feature vector.
Given a DNA sequence D (**Eq. 3**), the PC-PseTNC feature vector of D is defined:

$$\mathbf{D} = [d_1 \; d_2 \; \cdots \; d_{64} \; d_{64+1} \; \cdots \; d_{64+\lambda}]^{\mathrm{T}} \tag{24}$$

where

$$d_k = \begin{cases} \dfrac{f_k}{\sum_{i=1}^{64} f_i + w\sum_{j=1}^{\lambda}\theta_j} & (1 \le k \le 64) \\[2ex] \dfrac{w\theta_{k-64}}{\sum_{i=1}^{64} f_i + w\sum_{j=1}^{\lambda}\theta_j} & (65 \le k \le 64+\lambda) \end{cases} \tag{25}$$

where f_k（k=1,2,···,64) is the normalized occurrence frequency of trinucleotide in the DNA sequence; the parameter λ is an integer, representing the highest counted rank (or tier) of the correlation along a DNA sequence; w is the weight factor ranged from 0 to 1; θ_j (j=1,2,···, λ) is called the j-tier correlation factor that reflects the sequence order correlation between all the most contiguous trinucleotide along a DNA sequence, which is defined:

$$\begin{cases} \theta_1 = \dfrac{1}{L\text{-}3}\sum_{i=1}^{L-3}\Theta(\mathrm{R}_i\mathrm{R}_{i+1}\mathrm{R}_{i+2}, \mathrm{R}_{i+1}\mathrm{R}_{i+2}\mathrm{R}_{i+3}) \\ \theta_2 = \dfrac{1}{L\text{-}4}\sum_{i=1}^{L-4}\Theta(\mathrm{R}_i\mathrm{R}_{i+1}\mathrm{R}_{i+2}, \mathrm{R}_{i+2}\mathrm{R}_{i+3}\mathrm{R}_{i+4}) \\ \theta_3 = \dfrac{1}{L\text{-}5}\sum_{i=1}^{L-5}\Theta(\mathrm{R}_i\mathrm{R}_{i+1}\mathrm{R}_{i+2}, \mathrm{R}_{i+3}\mathrm{R}_{i+4}\mathrm{R}_{i+5}) \qquad (\lambda < L) \\ \qquad \cdots\cdots \\ \theta_\lambda = \dfrac{1}{L\text{-}2\text{-}\lambda}\sum_{i=1}^{L-2-\lambda}\Theta(\mathrm{R}_i\mathrm{R}_{i+1}\mathrm{R}_{i+2}, \mathrm{R}_{i+\lambda}\mathrm{R}_{i+\lambda+1}\mathrm{R}_{i+\lambda+2}) \end{cases} \tag{26}$$

where the correlation function is given by

$$\Theta(\mathrm{R}_i\mathrm{R}_{i+1}\mathrm{R}_{i+2}, \mathrm{R}_j\mathrm{R}_{j+1}\mathrm{R}_{j+2}) = \frac{1}{\mu}\sum_{u=1}^{\mu}[P_u(\mathrm{R}_i\mathrm{R}_{i+1}\mathrm{R}_{i+2}) - P_u(\mathrm{R}_j\mathrm{R}_{j+1}\mathrm{R}_{j+2})]^2 \tag{27}$$

where μ is the number of physiochemical indices (**Table 2**); $P_u(\mathrm{R}_i\mathrm{R}_{i+1}\mathrm{R}_{i+2})$ ($P_u(\mathrm{R}_j\mathrm{R}_{j+1}\mathrm{R}_{j+2})$) represents the numerical value of the u-th (u = 1, 2,… μ)

©2015 Computational Biology & Drug Design Group, CSU, China

physiochemical index for the trinucleotide $\mathrm{R}_i\mathrm{R}_{i+1}\mathrm{R}_{i+2}(\mathrm{R}_j\mathrm{R}_{j+1}\mathrm{R}_{j+2})$ at position $i(j)$.

For more information of PC-PseTNC approach, you can refer to (Chen, et al., 2014) (Qiu, et al., 2014)

The parameters:

- *lamada*: an integer larger than or equal to 0 and less than or equal to L-3 (L means the length of the shortest sequence in the dataset), representing the highest counted rank (or tier) of the correlation along a DNA sequence. The default value is 1.
- *w*: the weight factor ranged from 0 to 1, its default value is 0.05.
- *phyche_index*: the 12 built-in physicochemical indices (**Table 2**), which the users can choose. Its type should be a list.

3.5 Series correlation pseudo dinucleotide composition

SC-PseDNC is a variant of PC-PseDNC. Given a DNA sequence D (**Eq. 3**), the SCPseDNC feature vector of D is defined:

$$\mathbf{D} = [d_1\ d_2\ \cdots\ d_{16}\ d_{16+1}\ \cdots\ d_{16+\lambda}\ d_{16+\lambda+1}\ \cdots\ d_{16+\lambda\Lambda}]^{\mathrm{T}} \tag{28}$$

where

$$d_k = \begin{cases} \dfrac{f_k}{\sum_{i=1}^{16} f_i + w\sum_{j=1}^{\lambda}\theta_j} & (1 \le k \le 16) \\ \dfrac{w\theta_{k-16}}{\sum_{i=1}^{16} f_i + w\sum_{j=1}^{\lambda\Lambda}\theta_j} & (17 \le \mathrm{k} \le 16+\lambda\Lambda) \end{cases} \tag{29}$$

where f_k（k=1, 2, ⋯, 16) is the normalized occurrence frequency of dinucleotide in the DNA sequence; the parameter λ is an integer, representing the highest counted rank (or tier) of the correlation along a DNA sequence; w is the weight factor ranged from 0 to 1; Λ is the number of physicochemical indices; θj (j = 1, 2, ⋯, λ) is called the j-tier correlation factor that reflects the sequence-order correlation between all the most contiguous dinucleotides along a DNA sequence, which is defined:

©2015 Computational Biology & Drug Design Group, CSU, China

$$\begin{cases} \theta_1 = \frac{1}{L\text{-}3}\sum_{i=1}^{L-3} \mathrm{J}^1_{i,i+1} \\ \theta_2 = \frac{1}{L\text{-}3}\sum_{i=1}^{L-3} \mathrm{J}^2_{i,i+1} \\ \quad \cdots\cdots \\ \theta_\Lambda = \frac{1}{L\text{-}3}\sum_{i=1}^{L-3} \mathrm{J}^\Lambda_{i,i+1} \qquad \lambda < (\mathrm{L}-2) \\ \quad \cdots\cdots \\ \theta_{\lambda\Lambda-1} = \frac{1}{L\text{-}\lambda\text{-}2}\sum_{i=1}^{L-\lambda-2} \mathrm{J}^{\Lambda-1}_{i,i+\lambda} \\ \theta_{\lambda\Lambda} = \frac{1}{L\text{-}\lambda\text{-}2}\sum_{i=1}^{L-\lambda-2} \mathrm{J}^{\Lambda}_{i,i+\lambda} \end{cases} \tag{30}$$

The correlation function is given by

$$\begin{cases} \mathrm{J}^\zeta_{i,i+m} = P_u(\mathrm{R}_i\mathrm{R}_{i+1}) \cdot P_u(\mathrm{R}_{i+m}\mathrm{R}_{i+\mathrm{m}+1}) \\ \zeta = 1,2,\cdots,\Lambda;\ m=1,2,\cdots,\lambda;\ i=1,2,\cdots,\mathrm{L}\text{-}\lambda\text{-}2 \end{cases} \tag{31}$$

where μ is the number of total physiochemical indices (**Table 1**); $P_u(\mathrm{R}_i\mathrm{R}_{i+1})$ $(P_u(\mathrm{R}_j\mathrm{R}_{j+1}))$ represents the numerical value of the *u*-th (*u* = 1, 2,… μ) physiochemical index for the dinucleotide $\mathrm{R}_i\mathrm{R}_{i+1}$ ($\mathrm{R}_j\mathrm{R}_{j+1}$) at position *i*(*j*).
For more information of the SC-PseDNC, please refer to (Chen, et al., 2014).

The parameters:

- *lamada*: an integer larger than or equal to 0 and less than or equal to *L*-2 (*L* means the length of the shortest DNA sequence in the dataset), representing the highest counted rank (or tier) of the correlation along a DNA sequence. The default value is 1.
- *w*: the weight factor ranged from 0 to 1, the default value is 0.05.
- *phyche_index*: The 38 built-in physicochemical indices (**Table 1**), which the users can choose. Its type should be a list.

©2015 Computational Biology & Drug Design Group, CSU, China

3.6 Series correlation pseudo trinucleotide composition

SC-PseTNC is a variant of PC-PseTNC. Given a DNA sequence D (Eq. 3), the SCPseTNC feature vector of D is defined:

$$\mathbf{D} = [d_1\ d_2\ \cdots\ d_{64}\ d_{64+1}\ \cdots\ d_{64+\lambda}\ d_{64+\lambda+1}\ \cdots\ d_{64+\lambda\Lambda}]^{\mathrm{T}} \tag{32}$$

where

$$d_k = \begin{cases} \dfrac{f_k}{\sum_{i=1}^{64} f_i + w\sum_{j=1}^{\lambda}\theta_j} (1 \le k \le 64) \\ \dfrac{w\theta_{k-64}}{\sum_{i=1}^{64} f_i + w\sum_{j=1}^{\lambda\Lambda}\theta_j} (65 \le k \le 64+\lambda\Lambda) \end{cases} \tag{33}$$

where f_k（k=1, 2, ···, 64) is the normalized occurrence frequency of trinucleotide in the DNA sequence; the parameter λ is an integer, representing the highest counted rank (or tier) of the correlation along a DNA sequence; w is the weight factor ranged from 0 to 1; Λ is the number of physicochemical indices; θ_j (j=1, 2, ···, λ) is called the j-tier correlation factor that reflects the sequence order correlation between all the most contiguous trinucleotides along a DNA sequence, which is defined:

$$\begin{cases} \theta_1 = \dfrac{1}{L\text{-}4}\sum_{i=1}^{L-4} \mathrm{J}_{i,i+1}^{1} \\ \theta_2 = \dfrac{1}{L\text{-}4}\sum_{i=1}^{L-4} \mathrm{J}_{i,i+1}^{2} \\ \cdots\cdots \\ \theta_\Lambda = \dfrac{1}{L\text{-}4}\sum_{i=1}^{L-4} \mathrm{J}_{i,i+1}^{\Lambda} \qquad \lambda < (\mathrm{L}-3) \\ \cdots\cdots \\ \theta_{\lambda\Lambda-1} = \dfrac{1}{L\text{-}\lambda\text{-}3}\sum_{i=1}^{L-\lambda-3} \mathrm{J}_{i,i+\lambda}^{\Lambda-1} \\ \theta_{\lambda\Lambda} = \dfrac{1}{L\text{-}\lambda\text{-}3}\sum_{i=1}^{L-\lambda-3} \mathrm{J}_{i,i+\lambda}^{\Lambda} \end{cases} \tag{34}$$

The correlation function is given by

$$\begin{cases} \mathrm{J}_{i,i+m}^{\zeta} = P_u(\mathrm{R}_i\mathrm{R}_{i+1}\mathrm{R}_{i+2}) \cdot P_u(\mathrm{R}_{i+m}\mathrm{R}_{i+\mathrm{m}+1}\mathrm{R}_{i+\mathrm{m}+2}) \\ \zeta = 1,2,\cdots,\Lambda;\ m=1,2,\cdots,\lambda;\ i=1,2,\cdots,\mathrm{L}\text{-}\lambda\text{-}3 \end{cases} \tag{35}$$

where μ is the number of physiochemical indices (**Table 2**); $P_u(\mathrm{R}_i\mathrm{R}_{i+1}\mathrm{R}_{i+2})$ ($P_u(\mathrm{R}_j\mathrm{R}_{j+1}\mathrm{R}_{j+2})$) represents the numerical value of the u-th (u = 1, 2,…μ) physiochemical index for the trinucleotide $\mathrm{R}_i\mathrm{R}_{i+1}\mathrm{R}_{i+2}$ ($\mathrm{R}_j\mathrm{R}_{j+1}\mathrm{R}_{j+2}$) at position i(j).

For more information of the SC-PseTNC approach, please refer to (Chen, et al., 2014)

The parameters:

- *lamada*: an integer larger than or equal to 0 and less than or equal to L-3 (L means the length of the shortest DNA sequence in the dataset), representing the highest counted rank (or tier) of the correlation along a DNA sequence. The default value is 1.

©2015 Computational Biology&Drug Design Group, CSU, China

- *w*: the weight factor ranged from 0 to 1, the default value is 0.05.
- *phyche_index*: the 12 built-in physicochemical indices (Table 2), which the users can choose. Its type should be a list.

4. Table 1

Base stacking	Protein induced deformability	B-DNA twist
A-philicity	Propeller twist	Duplex stability: (freeenergy)
DNA denaturation	Bending stiffness	Protein DNA twist
Aida_BA_transition	Breslauer_dG	Breslauer_dH
Electron_interaction	Hartman_trans_free_energy	Helix-Coil_transition
Lisser_BZ_transition	Polar_interaction	SantaLucia_dG
SantaLucia_dS	Sarai_flexibility	Stability
Sugimoto_dG	Sugimoto_dH	Sugimoto_dS
Duplex tability (disruptenergy)	Stabilising energy of Z-DNA	Breslauer_dS
Ivanov_BA_transition	SantaLucia_dH	Stacking_energy
Watson-Crick_interaction	Dinucleotide GC Content	Twist
Tilt	Roll	Shift
Slide	Rise	

Note: By now, 37 kinds of *phyche_index* in table 1 is available except "Duplex stability: (freeenergy)".

5.Table 2

Bendability (DNAse)	Bendability (consensus)	Trinucleotide GC Content
Consensus_roll	Consensus-Rigid	Dnase I
MW-Daltons	MW-kg	Nucleosome
Nucleosome positioning	Dnase I-Rigid	Nucleosome-Rigid

©2015 Computational Biology&Drug Design Group, CSU, China

6.Table 3

Twist	Tilt	Roll
Shift	Slide	Rise

©2015 Computational Biology&Drug Design Group,CSU,China
