# Supplementary material for: BioTriangle: a web-accessible platform for generating various molecular representations for chemicals, proteins, DNAs/RNAs and their interactions
Source: J Cheminform. 2016 Jun 21;8:34. doi: 10.1186/s13321-016-0146-2 (PMC4915156; doi:10.1186/s13321-016-0146-2)
Supplement: Supplementary file 4 — 10.1186/s13321-016-0146-2 Features of BioCCI, BioPPI and BioDDI. [file 13321_2016_146_MOESM4_ESM.pdf]

BioTriangle

--BioCCI, BioPPI, BioDDI

COMPUTATIONAL BIOLOGY &
DRUG DESIGN GROUP
CENTRAL SOUTH UNIV., CHINA

The descriptor calculation of chemical-chemical interaction, protein-protein interaction, and DNA/RNA-DNA/RNA interaction is similar to each other in BioCCI, BioPPI and BioDDI. Next, we will show how to construct an interaction feature by the protein-protein interaction example.

Protein-protein interaction descriptors

Let $\mathbf{F_a} = \{\mathbf{F_a}(i), i = 1, 2, \ldots, p\}$ and $\mathbf{F_b} = \{\mathbf{F_b}(i), i = 1, 2, \ldots, p\}$ are the two descriptor vectors for interaction protein A and protein B, respectively. There are three methods to construct the interaction descriptor vector **F** for A and B:

(1) Two vectors $\mathbf{F_{ab}}$ and $\mathbf{F_{ba}}$ with dimension of 2p are constructed: $\mathbf{F_{ab}} = (\mathbf{F_a}, \mathbf{F_b})$ for interaction between protein A and protein B and $\mathbf{F_{ba}} = (\mathbf{F_b}, \mathbf{F_a})$ for interaction between protein B and protein A.

(2) One vector **F** with dimension of 2p is constructed: $\mathbf{F} = \{\mathbf{F_a}(i) + \mathbf{F_b}(i), \mathbf{F_a}(i) \times \mathbf{F_b}(i), i = 1, 2, \ldots, p\}$.

(3) One vector **F** with dimension of p^2 is constructed by the tensor product: $\mathbf{F} = \{\mathbf{F}(k) = \mathbf{F_a}(i) \times \mathbf{F_b}(j), i = 1, 2, \ldots, p, j = 1, 2, \ldots, p, k = (i-1) \times p+j\}$.

©2015 Computational Biology & Drug Design Group, CSU, China
