# Supplementary material for: BioTriangle: a web-accessible platform for generating various molecular representations for chemicals, proteins, DNAs/RNAs and their interactions
Source: J Cheminform. 2016 Jun 21;8:34. doi: 10.1186/s13321-016-0146-2 (PMC4915156; doi:10.1186/s13321-016-0146-2)
Supplement: Supplementary file 5 — 10.1186/s13321-016-0146-2 Features of BioCPI, BioDPI and BioCDI. [file 13321_2016_146_MOESM5_ESM.pdf]

BioTriangle

--BioCPI, BioDPI, BioCDI

COMPUTATIONAL BIOLOGY &
DRUG DESIGN GROUP
CENTRAL SOUTH UNIV., CHINA

The descriptor calculation of chemical-protein interaction, protein-DNA/RNA interaction, and chemical-DNA/RNA interaction is similar to each other in BioCPI, BioDPI and BioCDI. Next, we will show how to construct an interaction feature by the chemical-protein interaction example.

Chemical-protein interaction descriptors

There are two methods for construction of descriptor vector **F** for chemical-protein interaction from the protein descriptor vector $\mathbf{F_t}$($\mathbf{F_t}$(i), i = 1, 2, ...,p_t) and chemical descriptor vector $\mathbf{F_d}$ ($\mathbf{F_d}$(i), i = 1, 2, ...,p_d)**:**

(1) One vector **V** with dimension of $p_t + p_d$ are constructed: **F** = ($\mathbf{F_t}$, $\mathbf{F_d}$)for interaction between protein T and ligand D.

(2) One vector **V** with dimension of $p_t \times p_d$ is constructed by the tensor product: **F** = {**F**(k) = $\mathbf{F_t}$(i) ×$\mathbf{F_d}$(j), i = 1, 2, …, p_t, j = 1, 2, …, p_d, k = (i-1) ×p_t+j}.

©2015 Computational Biology & Drug Design Group, CSU, China
